# Wnt/β-catenin signalling is necessary for gut differentiation in a marine annelid, *Platynereis dumerilii*

**DOI:** 10.1186/s13227-018-0100-7

**Published:** 2018-06-11

**Authors:** Radim Žídek, Ondřej Machoň, Zbyněk Kozmik

**Affiliations:** 1 Institute of Molecular Genetics, Czech Academy of Sciences, Vídeňská 1083, 142 20 Prague 4, Czech Republic; 2 Present Address: Institute of Experimental Medicine, Czech Academy of Sciences, Vídeňská 1083, 142 20 Prague 4, Czech Republic

**Keywords:** Tcf/LEF, C-clamp, HMG, Expression pattern, Protostomia, Polychaeta, Alternative splicing, Proteases, Proliferation, Gut development

## Abstract

**Background:**

Wnt/β-catenin (or canonical) signalling pathway activity is necessary and used independently several times for specification of vegetal fate and endoderm, gut differentiation, maintenance of epithelium in adult intestine and the development of gut-derived organs in various vertebrate and non-vertebrate organisms. However, its conservation in later stages of digestive tract development still remains questionable due to the lack of detailed data, mainly from Spiralia.

**Results:**

Here we characterize the *Pdu*-*Tcf* gene, a *Tcf/LEF* orthologue and a component of Wnt/β-catenin pathway from *Platynereis dumerilii,* a spiralian, marine annelid worm. *Pdu*-*Tcf* undergoes extensive alternative splicing in the C-terminal region of the gene generating as many as eight mRNA isoforms some of which differ in the presence or absence of a C-clamp domain which suggests a distinct DNA binding activity of individual protein variants. *Pdu*-*Tcf* is broadly expressed throughout development which is indicative of many functions. One of the most prominent domains that exhibits rather strong *Pdu*-*Tcf* expression is in the putative precursors of endodermal gut cells which are detected after 72 h post-fertilization (hpf). At day 5 post-fertilization (dpf), *Pdu*-*Tcf* is expressed in the hindgut and pharynx (foregut), whereas at 7 dpf stage, it is strongly transcribed in the now-cellularized midgut for the first time. In order to gain insight into the role of Wnt/β-catenin signalling, we disrupted its activity using pharmacological inhibitors between day 5 and 7 of development. The inhibition of Wnt/β-catenin signalling led to the loss of midgut marker genes *Subtilisin*-*1*, *Subtilisin*-*2*, *α*-*Amylase* and *Otx* along with a drop in β-catenin protein levels, *Axin* expression in the gut and nearly the complete loss of proliferative activity throughout the body of larva. At the same time, a hindgut marker gene *Legumain* was expanded to the midgut compartment under the same conditions.

**Conclusions:**

Our findings suggest that high Wnt/β-catenin signalling in the midgut might be necessary for proper differentiation of the endoderm to an epithelium capable of secreting digestive enzymes. Together, our data provide evidence for the role of Wnt/β-catenin signalling in gut differentiation in *Platynereis*.

## Background

Wnt/β-catenin signalling represents one of the most important and intensively studied signalling pathways in metazoan development. Posterior Wnt activity specifies the primary axis in early embryos with regulatory development or orients asymmetrical cell divisions and specifies vegetal cell fates in a similar way in organisms that exhibit a fixed stereotypical development [1]. Vegetal blastomeres usually give rise to the mesoderm and endoderm. For example, Wnt/β-catenin signalling is necessary for both the vegetal fate, midgut and hindgut specification in the sea urchin [2, 3] and is also indispensible for gastrodermal differentiation and maintenance in the sea anemone, *Nematostella vectensis* [4], suggesting an ancient role of Wnt/β-catenin signalling in endodermal and gut development. This also illustrates that Wnt/β-catenin can have two or more interconnected or independent roles in the subsequent stages of gut development [5] and homoeostasis [6] where it controls proliferation versus differentiation [7].

One of the most prominent examples is the mammalian intestine. A posterior source of Wnts is involved in the patterning of the primitive gut [5] where it directly activates *Cdx* genes in the hindgut [8] in both vertebrates [9–11] and insects [12]. Additionally, Wnt activity has also been observed on the villi of the developing gut [13]. On the other hand, in the large intestine of adult vertebrates, Wnt signal produced by Paneth cells on the bottom of intestinal crypts is received by neighbouring Lgr5+ stem cells [14]. In response, these cells divide asymmetrically to form progenitors and differentiated cells. Wnt/β-catenin signalling is thus crucial for the renewal of gut epithelium [6]. However, whether a similar role for Wnt/β-catenin signalling also exists in other bilaterian clades remains unclear since much less is known about the role of Wnt/β-catenin signalling in later gut development of protostomes.

In the Wnt/β-catenin pathway, which is active in the early specification of the endoderm and in gut development and maintenance, the transcriptional response is regulated by altering stability and thus the levels of β-catenin protein. In the absence of a Wnt signal, cytoplasmic β-catenin is phosphorylated by glycogen synthase kinase-3β (GSK-3β) in a so-called destruction complex that consists of Axin, adenomatous polyposis coli (APC) and the priming kinase casein kinase-1α (CK-1α). This leads to the ubiquitination of β-catenin by β-TrCP E3 ubiquitin ligase and rapid degradation of β-catenin in proteasomes. With low β-catenin, Tcf family transcription factors in the nucleus bind regulatory regions of target genes and repress transcription [15, 16].

The binding of the Wnt signal protein to an extracellular domain of a Frizzled family receptor ultimately leads to inactivation of the destruction complex. Hence, β-catenin is no longer degraded and can accumulate in the cytoplasm and nucleus, where it binds to a Tcf family transcription factors on the promoters of target genes and provides them with a transactivation domain which allows the activation or derepression of transcription. Levels of β-catenin can thus serve as a read-out of activity of this pathway. Furthermore, Axin is not only a member of the destruction complex, but also a direct Wnt target in mouse [17], human [18, 19] and zebrafish [20]; therefore, the amount of its transcripts corresponds to pathway activity.

There is only one orthologue of the Tcf gene in all Protostomia studied so far, with the exception of Platyhelminthes [16, 21] and Planaria [21] which have three or five *Tcf* genes, respectively. Most vertebrates possess four *Tcf/LEF* genes due to several rounds of whole-genome duplications: *Tcf1* (or *Tcf7*) [22], *LEF1* [23, 24], *Tcf3* (*Tcf7l1*) [25] and *Tcf4* (*Tcf7l2*) [26]. In *Xenopus*, Tcf3 functions as a transcriptional repressor in the absence of a Wnt signal and is replaced at promoters of target genes by Tcf1/β-catenin complex upon Wnt activation [27]. In general, Tcf3 is considered to function mostly as a repressor, Tcf1 and LEF1 as activators, while Tcf4 can function in either capacity, both in the absence or presence of a Wnt signal [28–30].

Tcf proteins contain an N-terminal β-catenin binding domain [31] which is followed by a GBS motif that is recognized by the Groucho co-repressor [32]. On the C terminus, there is a highly conserved HMG box which is followed by a basic tail of amino acids. They together constitute the HMG DNA binding domain (HMG DBD) that recognizes the specific DNA sequence in front of target genes [22, 23]. The basic tail serves as a nuclear localization signal [33] and also helps the HMG domain to bend the bound DNA [34, 35]. All invertebrate (except Planaria and Platyhelminthes) and some vertebrate Tcf genes also encode an auxiliary DNA binding domain that is C-terminal to HMG DBD and contains conserved cysteines, hence called a C-clamp. In vertebrates, only some isoforms of LEF1 and Tcf4 (called E isoforms) possess a C-clamp [36, 37]. C-clamps are thought to help HMG to select, restrict and strengthen binding to their target sites. Some genes, e.g. *Cdx1*, are only activated by isoforms of Tcf that possess a C-clamp [37].

*Platynereis dumerilii* is a marine polychaete annelid and an emerging spiralian animal model. Its development has been described elsewhere in much greater detail [38, 39]. In summary, *Platynereis* embryos undergo a fixed stereotypical development in which every cell has a defined fate which becomes more restricted with every round of embryonic cleavage. These cleavages are polarized by Wnt/β-catenin pathway activity vegetally in each cell/daughter cell pair [39]. The first three cleavages produce four vegetal macromeres as progenitors of the endoderm [39, 40]. Later, blastoporal lips encapsulate the large macromeres (yolk cells), each containing a lipid droplet. Yolk cells with gut precursors divide in a process called cellularization to form a gut cavity and the larvae start to feed between 5 and 7 days of development [38, 41]. Proliferation continues in the ring of stem cells between the last segment and the pygidium in the segment addition zone [42, 43].

Even though the expression of genes encoding for Wnt proteins, Frizzled receptors and the signal transduction protein Axin has been described in *Platynereis* to some extent, it has been done mostly during earlier stages of development [44–47]. Surveys of *Platynereis* Wnt genes revealed that it possess 12 out of 13 families of Wnts, with the exception of the “canonical” Wnt3 [45, 48]. They are expressed laterally in the episphere, along the blastopore, in developing and regenerating segments, the ventral midline, the pygidial/proctodeal area and the posterior growth zone [44, 45, 49]. Unfortunately, nothing is known about the expression of *Platynereis* Wnt genes in later larval stages or expression in the gut.

There are four Frizzled receptors of Wnt ligands on signal-receiving cells in *P. dumerilii*, three *Frizzled*-related genes, and two genes which encode for soluble Frizzled receptors [47] that inhibit Wnt/β-catenin signalling. In later development, from 3 to 5 dpf, *Pdu*-*Fz4* is first expressed in the brain and stomodeum, then later fades out and is more abundant in what appears to be the foregut/midgut and midgut/hindgut borders rather than the mesoderm as reported. *Pdu*-*Sfrp3/4* is restricted to small expression domains anteriorly to each parapodium at the same stage. However, the expression of other Frizzled-related genes was not examined in these stages.

In this paper, we identified a single *Platynereis dumerilii*’s *Tcf* orthologue, *Pdu*-*Tcf*, which generates an array of products via alternative splicing. We provide a description of its expression and focus on its function in the developing gut. We also report the presence of other components of Wnt/β-catenin signalling, namely *Pdu*-*Axin* and β-catenin protein, in the gut. We show that manipulation of the activity of Wnt/β-catenin signalling influences cell proliferation in the developing larva and affects the expression of endodermal markers and gut digestive enzymes, leading to changes in the functional division of the gut. We propose that active Wnt/β-catenin signalling is necessary for cell proliferation and proper differentiation of gut compartments.

## Methods

### Gene identification and cloning

We searched *P. dumerilii* EST databases with sequences of known *Tcf* homologues found in other organisms and found two contigs, 05083 and 02618, that exhibit a high similarity to the N or C terminus of *Tcf*, respectively. By PCR amplification, we obtained a complete cDNA lacking a C-clamp together with N-terminal probe for in situ hybridization.

The C-clamp (−) isoform, X7, of *Pdu*-*Tcf* cDNA was amplified by polymerase chain reaction using Long PCR Enzyme Mix (Thermo Fisher Scientific#K0182), forward primer TcfPlatyRT-1 (5′-GGGAGATTTTCATGGCGGATTCA-3′) and the reverse primer TcfPlatyRT-4 (5′-CAGTTAGATCAAGCAGAGGTCAGAAGTAATACC-3′) on mixed stage *Platynereis* cDNA as a template. cDNA was synthesized using SuperScript™ II RT (Invitrogen 18064014) and random hexamer primers (Invitrogen 48190-011) following the manufacturer’s protocol from mRNA isolated with TRIzol™ Reagent (Invitrogen 15596026) according to manufacturer’s instructions. Conditions for PCR were as follows: initial denaturation 95 °C/2 min followed by 30 cycles of 95 °C/20 s denaturation, 61 °C/30 s annealing and at 68 °C/3-min extension, closed by an additional extension period of 10 min. The resulting fragment was then cloned into pGEM T-Easy Vector System (Promega) and sequenced.

The N-terminal fragment of *Pdu*-*Tcf* utilized for probe synthesis was amplified using Pfu polymerase, together with the TcfPlatyRT1 forward primer with the tail that contained the EcoRI restriction site and the TcfPlaty RT-2 (5′-CTGTACAAGGGATGATGGAACTGGC-3′) + BamHI tail reverse primer. Fragment was then isolated on the gel and the included restriction sites digested by EcoRI and BamHI. Resulting overhangs were used to clone the fragment into the pBlueskript II KS vector and used for probe synthesis.

Sequencing revealed that this *Tcf* possessed a termination codon after the HMG DBD and thus lacked a C-terminal accessory DNA binding C-clamp domain. Since most of the protostomes to which *Platynereis* belongs have only one *Tcf* gene and out of these all possess a C-clamp domain [16, 21], we further searched TSA databases which yielded two more cDNA sequences, GBZT01001652.1 and GBZT01006558.1, from BioProject PRJNA271451 [50] It is of note that the former had a C-clamp and the latter a difference in the beginning of the HMG domain, but otherwise were found to be similar. We designed forward primers that were specific for two different variants of the 5′-HMG exon (5′-TGATGAGAACGAGGTGCAGGA-3′ and 5′-GACCACACACCCAATGATAGCG-3′) and the common reverse primer (5′-TCATAGTGGCGGTGGTTCCA-3′) after the C-clamp using the Primer3 online tool [51, 52]. Different C-terminal isoforms were amplified in two separate PCRs with AccuPrime™ *Pfx* SuperMix (Invitrogen Life Technologies Cat. No. 12344-040), separated by agarose gel electrophoresis, cloned into a pCR™-Blunt vector using a Zero Blunt™ PCR Cloning Kit (Invitrogen, Thermo Fisher Scientific K2750-20) and used for sequencing and probe synthesis.

There is a short 36nt sequence that corresponds to 12 amino acids of β-catenin binding domain after second intron, which is only facultatively included into the transcript. It is present in both available cDNA sequences from the closely related polychaete, *Perinereis nuntia* (NCBI GenBank accession numbers AB701688 and AB701687, [53]), and two *P. dumerilii* TSA sequences but not in another publicly available *P. dumerilii Tcf* sequence (NCBI GenBank number KT266551, Simon F., unpublished). No such sequence was found in other protostome sequences that we analysed, and an entirely different sequence was observed in deuterostomes. We thus used the N-terminal probe which excluded this variable region to assess the *Pdu*-*Tcf* expression patterns although we included this region in the phylogenetic analysis.

An *Axin* cDNA fragment was amplified and cloned using a publicly accessible sequence [46] with 5′-AGTTCCTCAATGACTCGGCA-3′ and 5′-CTTCCTGTAACGTGGGGAGT-3′ forward primer and reverse primers, respectively. The Axin fragment was amplified from mixed stage cDNA by AccuPrime™ *Pfx* SuperMix and cloned into a pCR™-Blunt vector using a Zero Blunt™ PCR Cloning Kit, verified by sequencing, and used to generate digoxigenin-labelled RNA probes.

Templates for probe synthesis of *Pdu*-*Subtilisin*-*1*, *Pdu*-*Subtilisin*-*2*, *Pdu*-*α*-*Amylase* and *Pdu*-*Legumain* were a gift from Gáspár Jékely’s laboratory [41]. Templates for *Pdu*-*Nk2.1* and *Pdu*-*Otx* probes were a gift from Detlev Arendt’s laboratory [54, 55]. *Pdu*-*Cdx* clone was obtained as a gift from David K. Ferrier [56].

### Protein alignment and molecular phylogeny

The protein alignment was done using BioEdit’s [57] ClustalW Multiple alignment and MEGA7’s [58] MUSCLE algorithms and improved manually. The GenBank accession numbers of the protein or translated nucleotide sequences used for comparison are as follows: *Perinereis nuntia* (AB701688.1), *Lingula anatina* (XP_013385963.1), *Crassostrea gigas* (XP_019923475.1), *Biomphalaria glabrata* (XP_013060932.1), *Limulus polyphemus* (XP_022255329.1), *Parasteatoda tepidariorum* (XP_021000199.1), *Drosophila melanogaster* (NP_001033798.1), *Tribolium castaneum* (XP_008191151.1), *Strongylocentrotus purpuratus* (NP_999640.1), *Sacoglossus kowalevskii* (XP_006811841.1), *Branchiostoma floridae* (AAZ77711.1), *Danio rerio* (NP_571334.1), *Xenopus laevis* (XP_018082716.1), *Anolis carolinensis* (XP_008112949.1), *Gallus gallus* (XP_015144041.1), *Homo sapiens* (NP_001185456.1). Pdu-Tcf protein sequence is an in silico translation of the longest *Pdu*-*Tcf* cDNA which we have identified (C-terminal isoform X1, Fig. 2c) obtained by merging the X1 C-terminal sequence with the full-length cDNA of the C-clamp (−) isoform X7 (Fig. 2c).

Molecular phylogeny was determined using MEGA7 software [58]. The evolutionary history was inferred by using the maximum likelihood method based on the JTT matrix-based model [59]. Initial trees for the heuristic search were obtained automatically by applying neighbour-join and BioNJ algorithms to a matrix of pairwise distances estimated using the JTT model and then selecting the topology with superior log likelihood value. A discrete gamma distribution was used to model evolutionary rate differences among the sites (five categories + G, parameter = 0.8124). All positions with less than 85% site coverage were eliminated. That is, up to 15% alignment gaps, missing data and ambiguous bases were allowed at any position to include the whole HMG domain in the analysis, since two of the sequences (*Perinereis nuntia* and *Limulus polyphaemus*) were truncated. There were a total of 343 positions in the final dataset.

### Animal culture and spawnings

Larvae and adult worms were collected from our established *Platynereis* breeding facility at the Institute of Molecular Genetics of the Czech Academy of Sciences in Prague. Mature worms were mated in pairs in natural sea water (NSW) in glass containers. Several minutes after spawning, the worms were discarded, and as soon as fertilized eggs settled to the bottom, most of the volume in the containers was replaced with fresh NSW. The next day, we removed all poorly developing embryos from the bottom of the glass cylinder and kept only the healthy population which used cilia to swim close to the surface of the water column. We kept the developing larvae designated for experiments at 18 °C to ensure proper staging [38].

### Sample collection and fixation

Larvae of the desired stage were immobilized by the addition of 1/10–1/3 volume of 4% PFA (from 16% PFA, Electron Microscopy Sciences 50-980-487) in PTw (1.86 mM NaH_2_PO_4_, 8.41 mM Na_2_HPO_4_, 175 mM NaCl, 0.1% Tween20, pH 7.4) and collected by a pipette into a 2-ml microtube. After sedimentation of the larvae, the solution was discarded and replaced with 2 ml of 4% PFA/PTw. Samples were then incubated at room temperature for 2 h, rocking slowly, followed by three washes with PTw and dehydrated with two washes in 100% MetOH. Subsequently, fixed larvae were stored in 100% MetOH at − 20 °C. All solutions used were prepared using diethyl pyrocarbonate (DEPC, Sigma D5758), treated (1:1000) autoclaved deionized water and filtered through a 0.22-µm syringe filters (Merck Millipore SLGS033SS or SLGP033RS) or tissue culture filters (Corning 431097).

### Chemical treatment

We concentrated 5 dpf (days post-fertilization) old larvae from a single batch on a fine sieve and collected them using a Pasteur pipette. The whole batch was then divided into four experimental groups, each consisting of 7 ml, in a 6-cm Petri dish. Chemicals used for treatment (dissolved and stored in dimethyl sulphoxide, DMSO, Sigma) or corresponding amount of DMSO alone as a control were diluted in a volume of NSW to 1 ml in total and mixed with the larvae. We used CHIR99021 as an activator of the Wnt/β-catenin pathway (Biomedica) at 10 µM final concentration, since it works well on earlier (24–48 hpf) stages and higher concentrations often killed the larvae, and inhibitors JW55 (own stock, but available commercially) or IWR-1-endo (Merck Millipore 681669) at a 30 µM concentration. Petri dish with larvae was then incubated at 18 °C for 2 days and collected at 7 dpf stage.

### Immunohistochemistry

Immunological staining of the nervous system, cilia, and β-catenin was based on in situ hybridization protocol. Fixation, storage, re-hydration, permeabilization by proteinase K and subsequent washes with PTw were performed in the same manner as the in situ hybridization protocol (see further), followed by blocking in Blocking 1 buffer [39] and incubation with monoclonal anti-acetylated tubulin (1:1000, Sigma T 6793) and anti-β-catenin (1:100, Sigma C2206) primary antibodies in Blocking 1 at 4 °C, overnight, shaking on a nutator.

On the second day, the larvae were washed 3 × 15 and 4 × 30 min in PTw and incubated with Alexa Fluor^®^ 555 goat anti-mouse IgG (H + L) (1:500, Molecular Probes. A21422) and Alexa Fluor^®^ 647 goat anti-rabbit IgG (H + L) (1:500, Molecular Probes 21245) in Blocking 1, in the dark, overnight, shaking on a nutating mixer. Unbound antibodies were removed with several washes of PTw, and the larvae were then transferred via a series of dilutions of gradually increasing concentrations of 2,2′-thiodiethanol in PTw to 97% TDE and stored at 4 °C in the dark.

### In situ hybridization on *Platynereis* larvae

Digoxigenin-labelled RNA probes were synthesized using a DIG RNA Labeling Mix (Roche 1277073) from plasmid templates, linearized with the appropriate restriction enzymes, and purified with QIAprep Spin Miniprep Kit (Qiagen) following the manufacturer’s recommendation, diluted in deionized distilled water, and their concentration measured using the Qubit™ RNA Assay Kit. The probes were then stored at − 80 °C.

Visualization of gene expression by whole mount in situ hybridization was done according to previously published protocols with some minor modifications. Fixed dehydrated larvae stored at − 20 °C in 100% MetOH were rehydrated by washing at least 5 min per wash in decreasing dilution series (75, 50 and 25%, respectively) of MetOH in PTw (DEPC treated, filtered) without rocking at room temperature. After three washes in PTw, the larvae were permeabilized by incubation in freshly prepared 0.1 mg/ml proteinase K (Roche 03115828001) in PTw for 1 min for 24, 48 and 72 hpf larvae, 2–2.5 min for 5 dpf larvae and 2.5–3 min for 7 dpf larvae. Proteinase treatment was stopped using two washes (2.5–3 min) in 2 mg/ml glycine in PTw (prepared in advance and stored in freezer). The larvae were then re-fixed by slow rocking in 4% PFA/PTw for 30 min at room temperature. The fixative was then removed by washing five times in PTw (5 or more minutes each wash), followed by one 10-min wash in hybridization buffer [50% deionized formamide, 0.75 M NaCl, 85 µM sodium citrate, heparin 50 µg/ml, 0.25% Tween20, 1% sodium dodecyl sulphate and 50 µg/ml single-stranded DNA from salmon testes (Sigma D9156) in DEPC-treated deionized water]. After the addition of fresh hybridization buffer, larvae from one sample were divided into several groups for staining of different genes and pre-hybrized for 2–4 h at 63 °C in a thermal block with a cover. After replacement of the pre-hybridization solution with 50 µl of digoxigenin-labelled RNA probes, 2 ng/µl in hybridization buffer (denatured previously for 10 min at 90 °C), the samples were hybridized approximately for 16–18 h overnight in a thermal block at 63 °C.

The following day, probes were collected for reuse and replaced with 250 µl of plain hybridization buffer. After the larvae settled to the bottom of the tubes (10–15 min), another 15-min wash with 250 µl of Hyb-Mix or 0.5 ml of 2× SSCT/50% formamide (Sigma) followed. Subsequently, the samples were washed twice in 2× SSCT/50 formamide for 30 min, twice in 2× SSCT for 15 min and twice in 0.2× SSCT for 30 min. All solutions used from hybridization up to this step were prepared using DEPC-treated autoclaved deionized water and pre-heated to the hybridization temperature of 63 °C The samples were kept in a thermal block at the hybridization temperature throughout this portion of the experiment (even during exchange of solutions). After last SSCT wash, the samples were transferred back to room temperature and washed twice with RT/cold 1× PTw and blocked in 2% w/V Boehringer-Mannheim Blocking Reagent (Roche 11 096 176 001) in maleic acid buffer (0.1 M maleic acid, 0.05 M NaCl, pH 7.5) for 1 h at RT, slowly rocking. The larvae were then incubated with anti-digoxigenin-AP, Fab fragments from sheep (1:4000, Roche 11 093 274 910) and monoclonal anti-acetylated tubulin (1:1000, Sigma T 6793) at 4 °C overnight (the latter was only applied for fluorescent in situ hybridization) while shaking on a nutating mixer.

On the third day, the samples were washed three times with PTw (5–10 min each) and four times in PTw (30 min each). After the last wash, the larvae were stored overnight at 4 °C in PTw. The next morning, the samples were briefly (5–10 min) washed again with PTw.

In the case of fluorescent in situ hybridization, the larvae were washed twice with 100 mM Tris–Cl, pH 8.5, 0.2% Tween20, filtered through a 0.22-µm syringe filters (Merck Millipore), and stained on 4-well or 24-well plate in a Vector^®^ Blue Alkaline Phosphatase Substrate (Vector Laboratories SK-5300) solution which was prepared according to the manufacturer’s instructions with 100 mM Tris–Cl, pH 8.5, 0.2% Tween20. A signal was developed in the dark at room temperature while slowly rocking.

For bright-field microscopy, after washing the larvae in PTw, we washed them twice (10 min each wash) in alkaline phosphatase (AP) staining solution without MgCl_2_ (50 mM Tris–Cl, pH 9.5, 100 mM NaCl, 0.1% Tween20) followed by two washes with the same buffer supplemented with 50 mM MgCl_2_. All solutions were filtered through a 0.22-µm syringe filter (Merck Millipore). The staining was done in the AP staining solution with MgCl_2_, 1.65 µl NBT (Roche 11 383 213 001) and 1.65 µl BCIP (Roche 11 383 221 001) per ml. Stock solutions of NBT and BCIP were first centrifuged for 2 min at 14,100 rcf to pellet the precipitated material. A signal was developed in the dark, overnight at 4 °C.

In both cases, the larvae were checked regularly for a developing signal. Staining was stopped by transferring them back to microtubes and washed five times (about 10 min) in PTw.

For fluorescent in situ, larvae were blocked in a Blocking 1 solution [39] consisting of 4% sheep serum, 2 mg/ml bovine serum albumin and 0.1% dimethyl sulphoxide for 1 h at RT, rocking. They were then incubated overnight at 4 °C on nutating mixer in Blocking 1 solution with DAPI (4′,6-diamidino-2-phenylindole, Sigma, 1:1000) and Alexa Fluor^®^ 555 goat anti-mouse IgG (H + L) (1:500, Molecular Probes. A21422) secondary antibody. After three washes (15, 15 and 30 min) in PTw, the larvae were gradually transferred through 10-min washes in a dilution series of 33, 66 and 97% (two times) to 97% 2,2′-thiodiethanol (TDE; Sigma 166782) in PTw and stored at 4 °C in the dark for up to several weeks.

### In situ hybridization on adult tails

Adult worms were subjected to starvation for 2 days in order to empty their digestive tracts prior to dissection. They were then immobilized by the addition of 1 M MgCl_2_ to sea water with worms to the final concentration of 50 mM, which is substantially less than previously used by others [38, 40, 60] but still proved to be sufficient. The last 20–25 segments were cut, and worms were then returned to cultures to regenerate. The tails were fixed in 4% PFA/PTw (DEPC treated, filtered) overnight at room temperature, rocking, washed and dehydrated in the same way as larval samples and stored in methanol at − 20 °C.

Whole mount in situ hybridization was done using the same protocol that was used for larvae with following modifications (as described in [60]): proteinase K treatment was prolonged to 10 min, and the specimens in the pre-hybridization buffer were heated to 80 °C for 30 min prior to hybridization to inactivate endogenous phosphatase activity. The hybridization was done at 63 °C for 90 h. Primary antibodies used were anti-digoxigenin-AP, Fab fragments from sheep (1:4000, Roche 11 093 274 910) and anti-β-catenin (1:100, Sigma C2206). Fluorescent in situ staining was done using Vector^®^ Blue followed by staining with DAPI together with Alexa Fluor^®^ 555 goat anti-mouse IgG (H + L) (1:500, Molecular Probes. A21422) secondary antibody. Stained samples were transferred to and stored in 97% TDE.

### Embedding and sectioning

Larvae stained with NBT/BCIP for *Pdu*-*Tcf* were washed 1× with distilled water, 1× in 70% EtOH for 30 s and 100% ethanol for 1 min. After the ethanol had been removed, it was replaced with 400 µl of Spurr low viscosity embedding resin (Sigma EM0300, prepared according to the manufacturer’s instructions) and incubated for at least 20 min while gently rocking. They were then placed into moulds filled with Spurr resin and left for 2 h at room temperature. The larvae were positioned and oriented within the moulds and placed at 72 °C overnight. Blocks were then sectioned to 4-µm thin sections.

### EdU labelling of proliferating cells

Chemical treatment with Wnt/β-catenin pathway activator or inhibitors was done from 5 dpf as described. At 6 dpf, 5-ethynyl-2′-deoxyuridine was added to the water to a 20 µM final concentration so it could be incorporated into the DNA of replicating cells until 7 dpf when the larvae would be fixed by 4% PFA/PTw and stored in 100% methanol. Proliferating cells were detected using Click-iT^®^ EdU Alexa Fluor^®^ 594 Imaging Kit (Invitrogen, C10339) according to the manufacturer’s instructions, followed by DAPI staining of nuclei overnight. Finally, the larvae were transferred gradually to 97% TDE mounting medium for confocal fluorescence microscopy.

### TUNEL detection of cell death

Larvae treated with Wnt/β-catenin pathway activator or inhibitors between days 5 and 7 of development were fixed and stored in 100% methanol. Dead or dying cells were detected using Click-iT™ TUNEL Alexa Fluor™ 488 Imaging Kit (Invitrogen, C10245) and nuclei counterstained with Hoechst 33342 following the kit protocol. After counterstaining, the larvae were gradually transferred to 97% TDE in PTw.

### Microinjections

Fertilized eggs of *P. dumerilii* were washed thoroughly with half a litre of filtered natural sea water (FNSW), treated with 0.1 mg/ml proteinase K in FNSW for 25 s to permeabilize the eggshell and then rinsed quickly with half a litre of FNSW. The zygotes were microinjected with the mixture of 0.4 µg/µl SuperTOPFlash-tdTomato (courtesy of Vladimir Korinek) and 1:5 Fast Green FCF dye. SuperTOPFlash-tdTomato carries the gene for tdTomato fluorescent protein under a promoter with 8 Tcf/LEF binding sites, which makes it a reporter that is responsive to Wnt/β-catenin signalling [61].

### Image acquisition, processing and quantification

Stained larvae were placed on a glass slide with three layers of Scotch tape (approximate thickness of each layer was 50 µm) as spacers in 80 µl of 97% TDE as a mounting medium which has the same refractive index as glass and low photobleaching [62]. Immunofluorescent and fluorescent in situ hybridization images were taken using a Leica TCS SP5 AOBS Tandem with LP/-/C HC PL APO 40×/1.30 OIL CS2 or LP/0.14-0.20/D HC PL APO 63×/1.40 OIL objective lenses and with Leica TCS SP8 microscopes with a APO 40×/1.30 OIL CS2 objective as *z*-stacks with a *z*-step of 0.42- and 0.42-µm pixel size resulting in cubic voxels. The appropriate wavelengths for excitation (633 or 635 µm for Vector Blue) and emission detection (720–800 µm for Vector Blue) and *z*-compensation of laser intensity and detector gain to compensate for signal loss with increasing depth in the sample were used. EdU and TUNEL stainings were imaged in Fusion software with 0.45 µm voxel size by Zyla 4.2 PLUS sCMOS camera (Andor) and the Dragonfly 503 spinning-disc confocal system (Andor) mounted on a Leica DMi8 core with HC PL APO 40×/1.30 OIL CS objective lens.

Brightness and contrast were adjusted linearly and uniformly in the same way for all *z*-stacks. Maximum *z*-projections (for β-catenin, EdU and TUNEL stainings) or 3D reconstructions (for in situ hybridization) were done with FIJI software (in the latter case using its 3D Viewer plug-in). Images were cropped and resized in FastStone Image Browser, and the figures were assembled in Adobe Illutrator CS4. Proliferating cells marked by EdU incorporation and dead/dying cells marked by TUNEL staining were counted manually on maximal projections of whole *z*-stacks using the Cell Counter plug-in in FIJI. The differences between treatments were evaluated by a Student’s *t*-test.

Bright-field images and composite images of bright field and fluorescence were taken on a Nikon Diaphot 300 inverted microscope with DIC optics by Canon EOS1100D camera and utilizing the Canon EOS Utility software Remote Shooting function. We took a bright-field image from every individual after in situ hybridization and assigned them to categories according to the expression in the gut to quantify the effect of chemical treatment.

## Results

### *Pdu-Tcf* is spliced into multiple isoforms

**Fig. 1:**
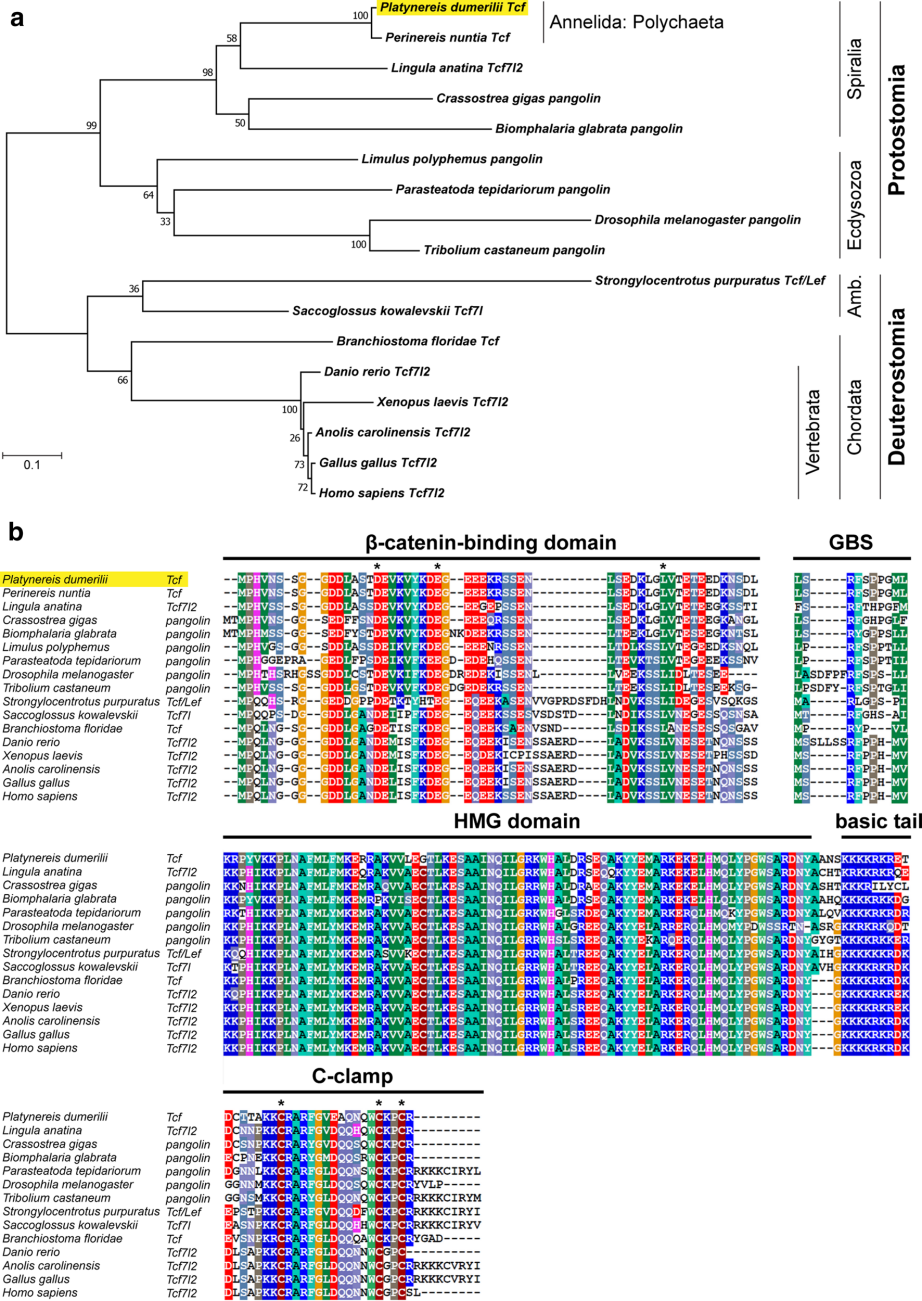
Phylogenetic relationships and conserved protein domains of *Pdu*-*Tcf.* **a** Phylogenetic analysis of Pdu-Tcf protein (shaded in yellow) sequence with top BLAST hits from selected organisms. The tree reveals that Pdu-Tcf clusters together with another polychaete *Perinereis nuntia* Tcf to the spiralian lineage of bilaterian Tcf sequences and is most similar to Tcf7l2 from the taxa where more *Tcf* genes are present. Protein sequences from taxa where we did not detect a β-catenin binding domain in addition to a HMG DNA binding domain using Pfam [63, 98], though their function as Tcfs was sometimes verified experimentally (e.g. *Caenorhabditis elegans* POP-1), were excluded from the analysis. The higher-order taxa are indicated on the right; Amb. = Ambulacraria. The tree with the highest log likelihood (− 6465.74) is shown. The percentage of trees in which the associated taxa clustered together is shown next to the branches. The tree is drawn to scale, with branch lengths measured in the number of substitutions per site. **b** Alignment of Pdu-Tcf with Tcf proteins known from other taxa shows the conservation of core domains necessary for Tcf function—N-terminal β-catenin binding domain, GBS—Groucho binding sequence, HMG box DNA binding domain with basic tail and the C-terminal C-clamp accessory DNA binding domain, characterised by the presence of the CRARF(Y) amino acid sequence. Asterisks denote conserved acidic amino acid residues within the β-catenin binding domain and cysteines within the C-clamp domains. Sequences which were incomplete (*P. nuntia* and *L. polyphemus* for HMG domain) or lacked the domain (*Xenopus laevis* for C-clamp) were excluded from the alignment. The extent of highlighted domains corresponds to those used previously by others [16, 21]

By BLAST search of EST databases with known sequences from other organisms, we have identified and cloned a homologue of a Tcf/LEF family transcription factor in the marine polychaete annelid *Platynereis dumerilii*. Phylogenetic analysis (Fig. 1a) confirmed that Pdu-Tcf represents a Tcf homologue which is the most similar to that of vertebrate Tcf7l2. Alignment of Pdu-Tcf with selected Tcf protein sequences from other organisms (Fig. 1b) shows that besides the HMG domain and a C-clamp, there is also a conserved N-terminal β-catenin binding domain (amino acids 1–166 as determined by Pfam [63]), GBS Groucho binding sequence and a basic tail, the latter of which immediately follows the HMG domain. *Pdu*-*Tcf* thus possesses all the necessary functional domains that are present in other organisms and required for the full spectrum of Tcf functions.

**Fig. 2:**
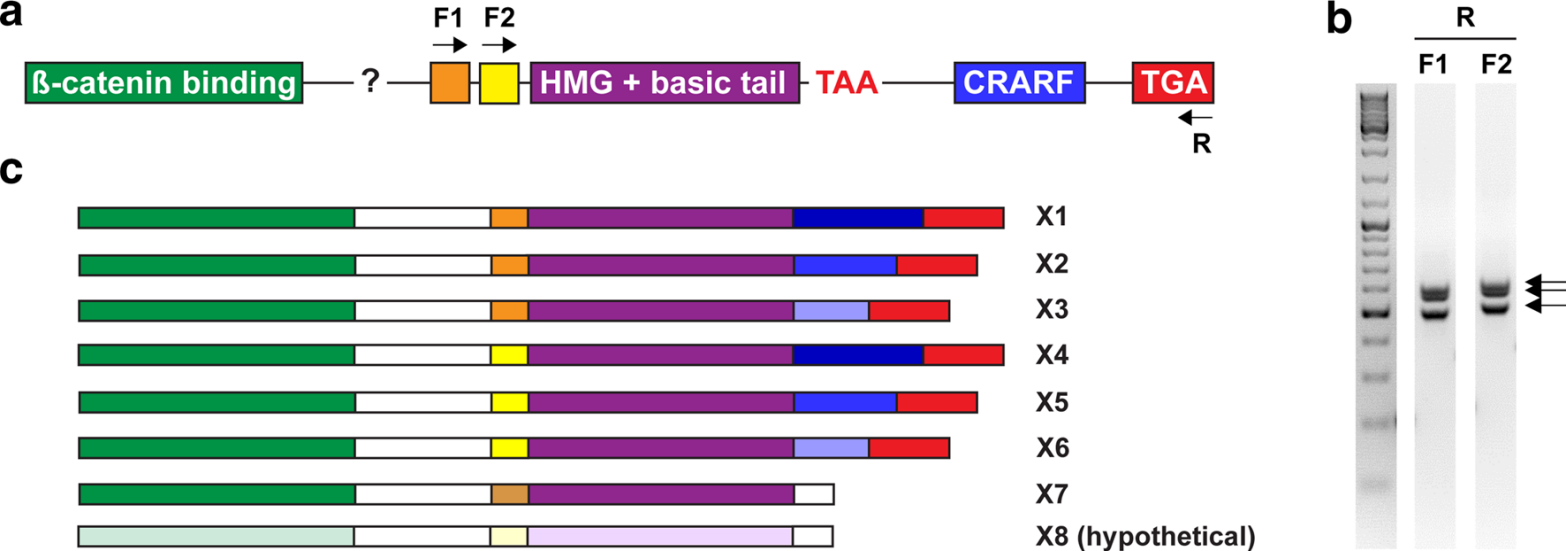
A single *Platynereis Tcf* gene produces multiple isoforms. **a** Known exon structure of *Pdu*-*Tcf* C terminus; N-terminal β-catenin binding domain is composed of at least 3 exons; two alternative exons for the beginning of HMG DNA binding domain, a common exon for most of the HMG domain, a premature stop codon within the intron following the HMG exon, alternative splice sites within a single C-clamp (CRARF) exon and a common C-terminal exon. Position of primers used to isolate the individual *Pdu*-*Tcf* isoforms is indicated. **b** The result of PCR on the cDNA template with the primers indicated in (**a**). Arrows point at three bands corresponding to three different splice variants amplified in each PCR. **c** Graphic representation of identified *Pdu*-*Tcf* isoforms in relation to their C-termini. We did not isolate the isoform X8, and we saw the isoform X6 only as a PCR product on gel. Note that these are isoforms which are defined only according to their C-termini and do not show the potential diversity of exons and alternative splicing in between the N-terminal β-catenin binding domain-encoding exon and C-terminal exons. Thus, each isoform listed here can actually form two or more variants

We designed forward primers that were specific to both variants of HMG domain and used them with the same reverse primer that was specific to the very end of the C-clamp (+) variant on mixed stage cDNA which was used as a template (Fig. 2a). We observed three products of slightly different sizes for each combination, showing that both HMG variants can combine with C-clamp of the same gene and that there are three variants of C-termini of a different length (Fig. 2b). We used the same combination of primers with genomic DNA to find the source of this variability. Sequencing of the amplified fragment revealed two alternative exons for the HMG domain and only one for C-clamp domain with several potential splice sites. In various C-clamp (+) variants, different splice sites are used, resulting in C-clamp domains of different lengths, which are then freely combined with either of the HMG variants giving rise to six different *Pdu*-*Tcf* isoforms (marked here as X1–X6, Fig. 2c) with respect to its C terminus.

Moreover, the end of C-clamp (−) Tcf is encoded by an intron of the same gene, directly following the basic tail and containing a cryptic termination codon. When the splice site is skipped and the intron retained, this stop codon prematurely terminates translation, resulting in a shorter protein that does not contain a C-clamp but does have the both β-catenin binding and HMG DNA binding domain, forming a potentially functional transcription factor of yet another isoform (here named X7). Although we observed C-clamp (−) C terminus in combination with only one HMG variant, we cannot rule out that it can also combine with the another one to making a putative isoform, X8. In summary, it appears that, like most protostomes, *Platynereis dumerilii* has only one Tcf gene and a diversity of isoforms is produced by alternative mRNA splicing.

### Expression of *Pdu-Tcf*

In order to get an overall view of the *Pdu*-*Tcf* and Wnt/β-catenin signalling’s role in *Platynereis* development, we performed whole mount in situ hybridization with an antisense digoxigenin-labelled probe that was complementary to the N-terminal part of *Pdu*-*Tcf* mRNA, which is common to and should hence detect all isoforms. We investigated *Pdu*-*Tcf* expression at 24, 48 and 72 hpf and 5 and 7 dpf larvae as well as adult worms.

**Fig. 3:**
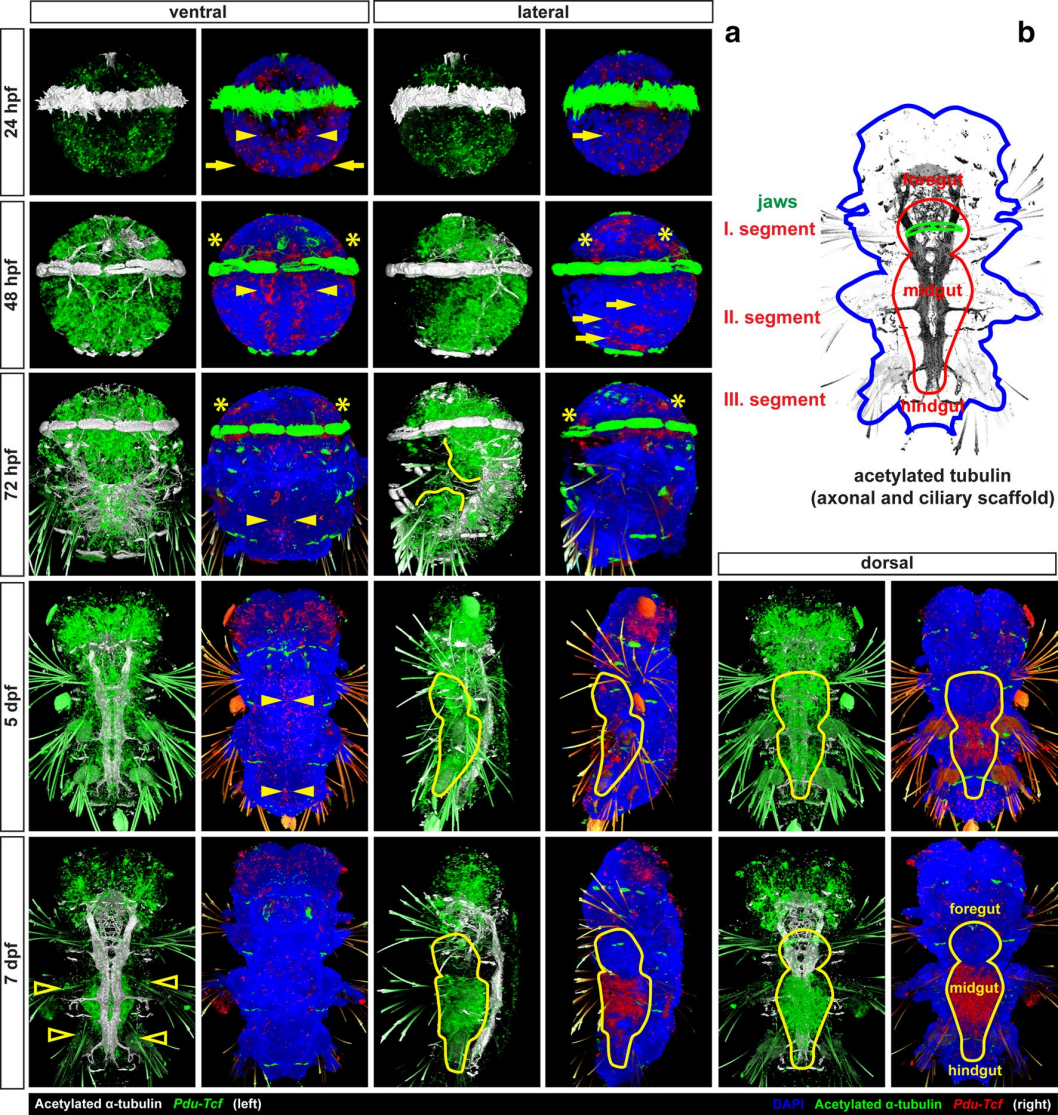
Expression of *Pdu*-*Tcf* throughout development of *Platynereis dumerilii.* **a** The expression of *Pdu*-*Tcf* during development (left: *Tcf* green, acetylated tubulin white; right: *Tcf* red, DAPI blue, acetylated tubulin green). At 24 hpf, *Pdu*-*Tcf* is broadly expressed at low levels in both the episphere and hyposphere. At 48 hpf in the hyposphere, *Pdu*-*Tcf* is present in ectodermal cells along the midline (yellow arrowheads) and in the segmental pattern (yellow arrows). It persists in the episphere, e.g. in future larval (ventral) and adult (dorsal) eye regions (yellow asterisks). At 72 hpf stage, *Pdu*-*Tcf* is expressed mainly in the episphere and stomodeal region, whereas it becomes more scarce in a majority of the hyposphere. This trend continues throughout 5 dpf, where expression mainly restricted to the brain ganglia of the head lobes. At 7 dpf, *Pdu*-*Tcf* is still expressed in the brain; however, a new strong expression is observed in the midgut and hindgut. There is also a small patch of weaker expression at the base of each parapodium (empty arrowheads). The expression patterns are described in greater detail in the text. Approximate size of a 48 hpf larva is around 130 μm, and all images are to scale. Stage and orientation are indicated; anterior up; in lateral view ventral to the right

At 24 hpf (Fig. 3a, top row), *Pdu*-*Tcf* was expressed at quite low levels broadly in both episphere (the upper half of the larva above the ciliary belt) and hyposphere (the lower half of the larva below the ciliary belt), except the area around the forming stomodaeum/blastoporus. In the episphere, it was detected slightly more laterally, while it was more abundant in ventrolateral region (neuroectoderm) of the hyposphere.

**Fig. 4:**
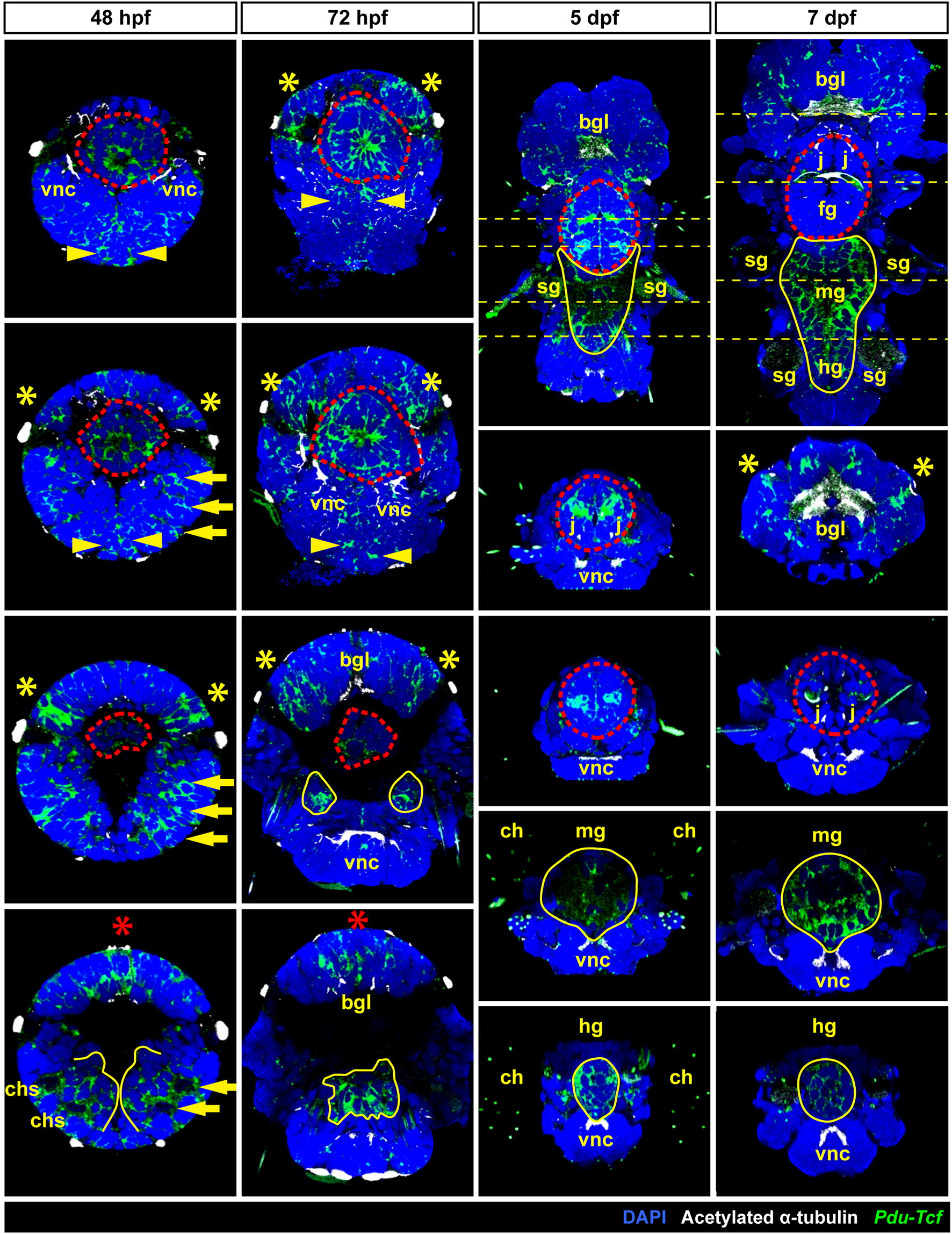
Analysis of *Pdu*-*Tcf* expression. Images show virtual orthogonal sections through confocal fluorescent *z*-stacks of *Pdu*-*Tcf* in situ hybridization staining to illustrate inner features and expression domains. The stages are indicated; 48 and 72 hpf—coronal sections from ventral to dorsal; 5 and 7 dpf, top—coronal sections with the positions of transverse sections indicated; 5 and 7 dpf, from top to bottom—consecutive transverse sections from anterior to posterior as indicated by a yellow dashed line on the coronal sections. The approximate size of a 48 hpf larva is around 130 µm, and other images are to scale. Red asterisk—apical organ, red dashed line—stomodaeal rosette/pharynx (foregut), yellow arrow—segmental expression in chaetal sacs, yellow asterisk—eye-forming region, yellow dashed line—position of transverse sections, yellow line—midgut + hindgut or putative gut tissue. *bgl* brain ganglia, *ch* chaetae, *chs* chaetal sacs, *fg* foregut, *hg* hindgut, *j* jaws, *mg* midgut, *sg* spinning glands, *vnc* ventral nerve cord(s)

At 48 hpf (Fig. 3a, second row), the expression of *Pdu*-*Tcf* was narrowed to more distinct domains, namely ectodermal segmental pattern consistent with the suggested role of Wnt/β-catenin signalling in segment formation [45, 49] and two stripes of ectodermal cells abutting the ventral midline. This domain probably corresponds to axin-expressing proliferating cells which were described by Demilly et al. [46] where it has been documented to respond to the Wnt signal produced by ventral midline. Also the presence of *Pdu*-*Tcf* in the stomodaeal rosette is quite strong. Ectodermal expression was also present in the apical region, where Wnt/β-catenin signalling has been suggested to regulate apical plate/apical organ development [64]. In the episphere, it can be found in ventrolateral and dorsolateral patches of ectoderm, the putative larval and adult eye regions, respectively. Moreover, *Pdu*-*Tcf* has also been observed in the internal segmental mesoderm of chaetal sacs, where also multiple Wnts are present [44] and in putative endodermal cells (Fig. 4, first column).

In 72 hpf larvae (Fig. 3a, third row), the expression persisted in the episphere and the stomodaeal region and it was retained, though weaker, in the two longitudinal stripes abutting ventral midline and diminished in segments. We detected notable delimitated expression domains in two bilaterally symmetrical clusters of cells internally in the larva between the second and third segment and probably continued within the larger central domain located more dorsally (Fig. 4, second column).

Five dpf larvae (Fig. 3a, fourth row) exhibit the strongest expression in brain ganglia. In the stomodaeum, it was expressed in two pairs of bilaterally symmetrical domains, one of which comprised the developing jaws. Weak expression was seen throughout the body, including the ventral midline, spinning glands and developing midgut, with higher expression observed in the hindgut (Fig. 4, third column)

**Fig. 5:**
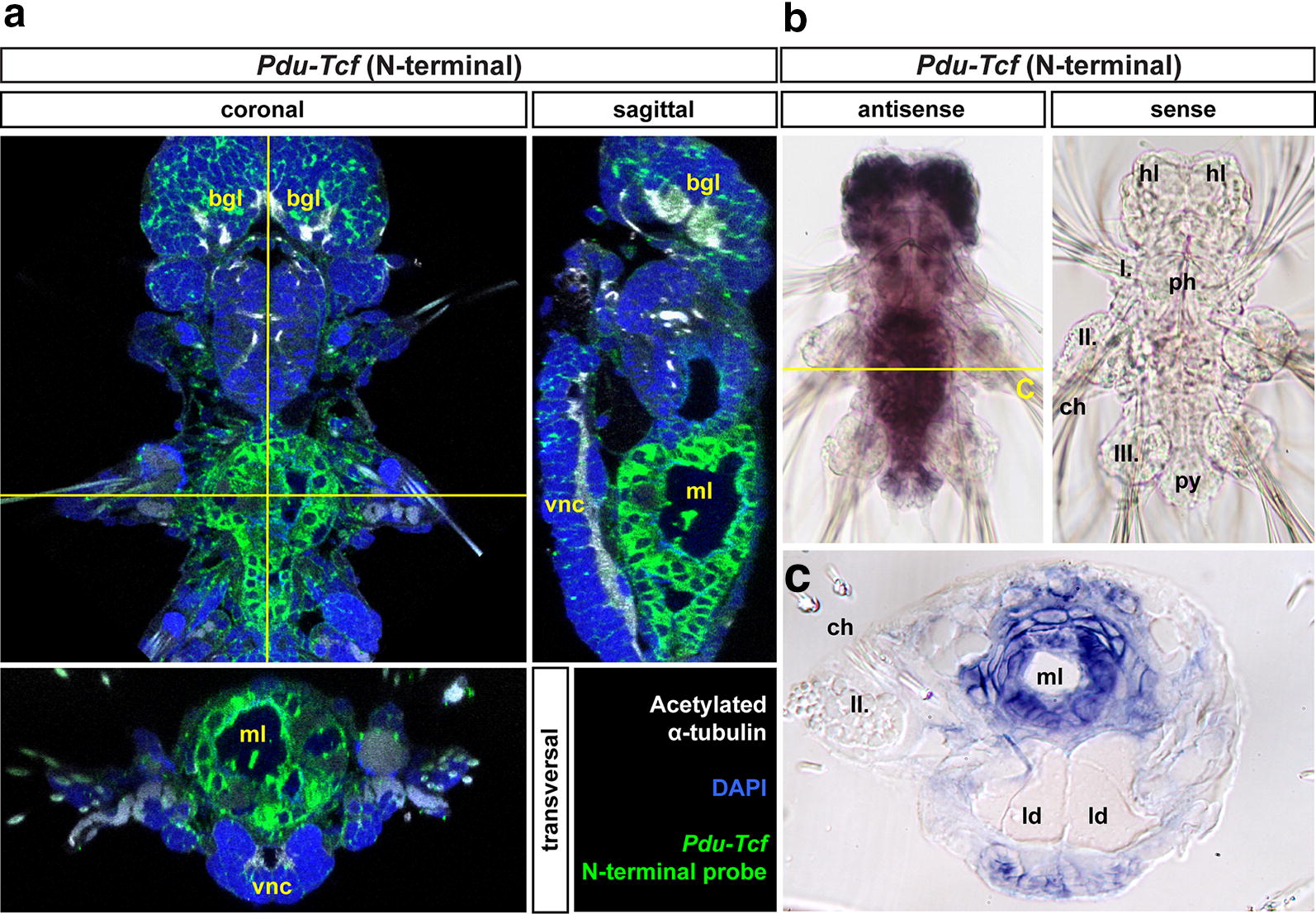
*Pdu*-*Tcf* is specifically detected in the gut of 7 dpf larva by N-terminal probe. **a** Virtual orthogonal sections through a confocal *z*-stack of a 7 dpf *Platynereis* larva after prolonged staining of fluorescent in situ hybridizaton with a probe recognizing the N-terminal part of *Pdu*-*Tcf* show the expression of this gene in the midgut and hindgut. **b** *Pdu*-*T*cf N-terminal antisense (left) and sense (right) probe NBT/BCIP stainings show that the antisense probe specifically detects *Pdu*-*Tcf* in the brain and in the gut. Also treatment with levamisole did not abolish the antisense probe staining demonstrating that the observed signal was not due to persisting endogenous alkaline phosphatase activity (which was thus successfully inactivated by hybridization temperature). The same fact is also demonstrated by the lack of staining with sense probe (right) or after hybridization without a probe (not shown). The absence of signal with sense probe shows that the observed signal is not due to unspecific binding of digoxigenin-labelled probe. **c** Physical coronal section through the body of 7 dpf larva after in situ hybridization with antisense probe against N terminus of *Pdu*-*Tcf* confirms its presence in the gut. The section is approximately on the level of the second segment, i.e. through midgut. I., II., III.—first, second and third body segments’ parapodia, *bgl* brain ganglia, *hl* head lobes, *ld* lipid droplets, *ml* midgut lumen, *ph* pharynx (foregut), *py* pygidium, *vnc* ventral nerve cord. For schematics of 7 dpf larval gut morphology, see Fig. 2

At 7 dpf (Fig. 3a, bottom row), the most prominent expression domain is in the midgut (Fig. 5), while it is still present in the hindgut, as revealed by prolonged staining (Fig. 5a), although in relatively lower level (Fig. 4—fourth column). Besides gut, *Pdu*-*Tcf* signal still remained high in the brain ganglia, and within sensory organs of the head lobes, it was present, though weaker, in the jaws, while it relatively decreased in the rest of the body. Small patches of cells exhibited the *Tcf* signal at the base of the second and third pair of parapodia and likely correspond to segmental ganglia. High *Pdu*-*Tcf* expression in the gut was intriguing because Wnt/β-catenin signalling in gut has not been previously reported in *Platynereis* and is also reminiscent of the crucial role of Wnt/β-catenin in gut development and maintenance of other organisms [5–7, 13]. The staining was specific since the sense probe produced no staining (Fig. 5b—right), while levamisole (an inhibitor of endogenous phosphatase, which is present in the larval gut [65]) added to samples during in situ protocol with an antisense probe did not alter staining (not shown).

**Fig. 6:**
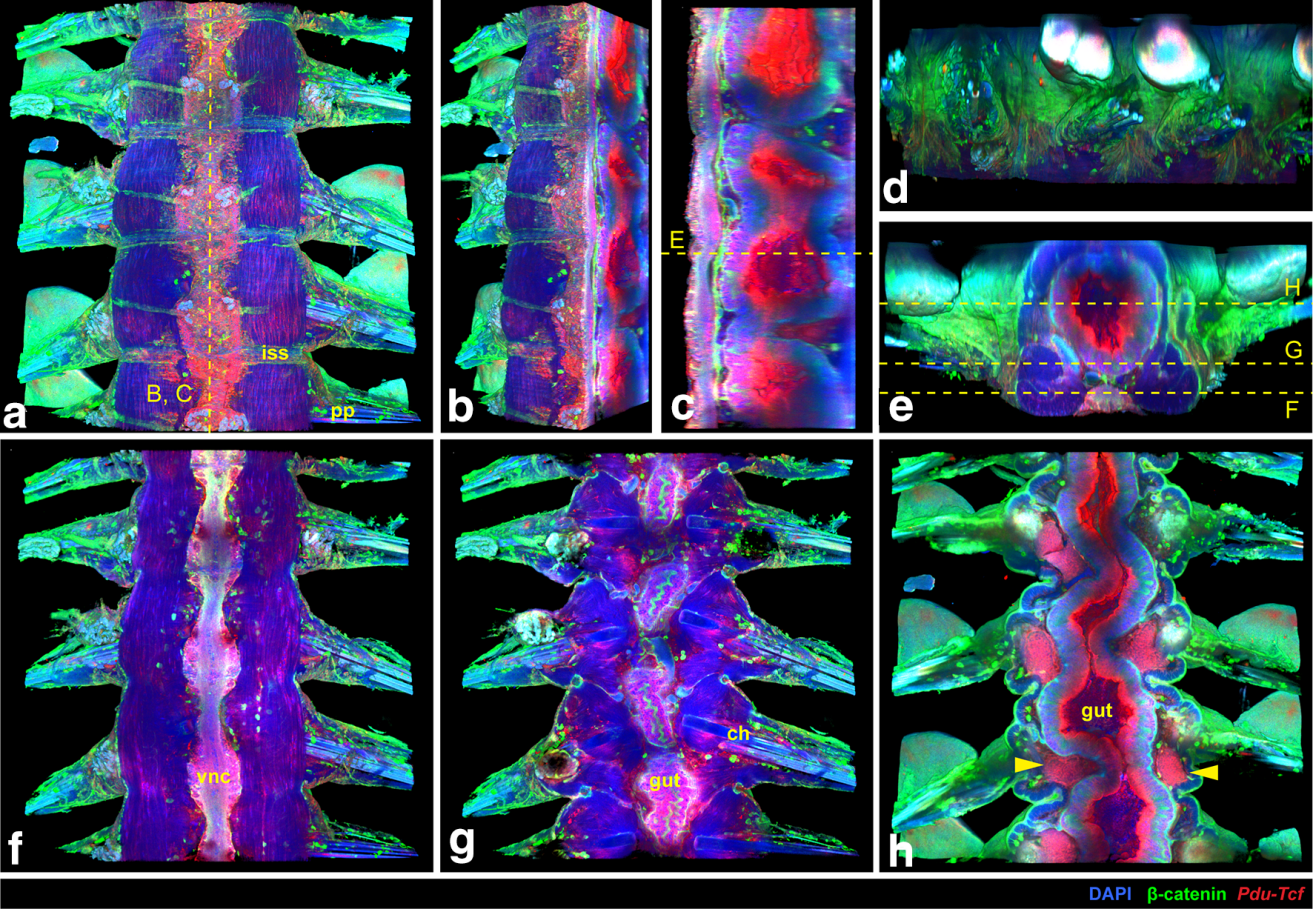
Expression of *Pdu*-*Tcf* in the adult worm. **a** 3D reconstruction of a confocal *z*-stack of several adult *Platynereis* trunk to tail segments showing the expression of *Pdu*-*Tcf* by fluorescent in situ hybridization (red) together with DAPI (blue) and β-catenin immunostaining (green) to visualize cell nuclei and surface; ventral view, anterior up. Sagittal section of the *z*-stack from (**a**) through the midline from ventrolateral (**b**) or lateral (**c**) view, ventral to the left, anterior up. Strong *Pdu*-*Tcf* expression signal in the intestine and constrictions of the gut between segments are apparent. **d** Lateral view of the same *z*-stack; ventral down, anterior to the left. **e** Transverse section through the middle of segment marked in (**c**) with the planes of coronal sections in (**f**–**h**) indicated. **f** Coronal section of the *z*-stack from (**a**) on the level of the ventral nerve cord (VNC), as indicated in (**e**). Some in situ signal is present in the VNC and weakly in muscles. **g** Another coronal section deeper in the body through the intestinal wall. In situ signal can be seen in the gut and in few cells around the base of chatae. β-catenin staining is strongest on the surface of the body and the gut epithelium. **h** Coronal section through the middle of the gut demonstrates strong *Pdu*-*Tcf* intestinal expression, especially on the luminal side, and in the intersegmental clusters of mesodermal cells (marked by yellow arrowheads), most probably the excretory system. *ch* chaetae, *iss* intersegmental septum, *pp* parapodia, *vnc* ventral nerve cord

Next, we analysed the expression of *Pdu*-*Tcf* in amputated tails of adult worms (Fig. 6). We observed the strongest signal on the luminal side of the gut. *Pdu*-*Tcf* was also present in the ventral nerve cord, in groups of mesodermal cells, possibly spinning glands and/or excretory system and around the base of chaetae, which might correspond to the signal seen in spinning glands at 5 dpf or segmental ganglia at 7 dpf, respectively (Fig. 3a).

### Wnt/β-catenin signalling in the developing gut of *P. dumerilii*

According to the literature, *Platynereis* larvae start to feed on algae between 5 and 7 days of development suggesting that a functional gut is already present [38], and at 6 dpf, larvae possess a gut divided into foregut, midgut and hindgut and express digestive enzymes [41]. Based on our observations, at 5 dpf large macromeres with lipid droplets (“yolk”) still obscured the gut cavity and the cellularization occurred between 5 and 7 days of development, since guts of 7 dpf larvae already consist of many cells and possess gut cavity (Fig. 4, right column, Fig. 5a, c).

**Fig. 7:**
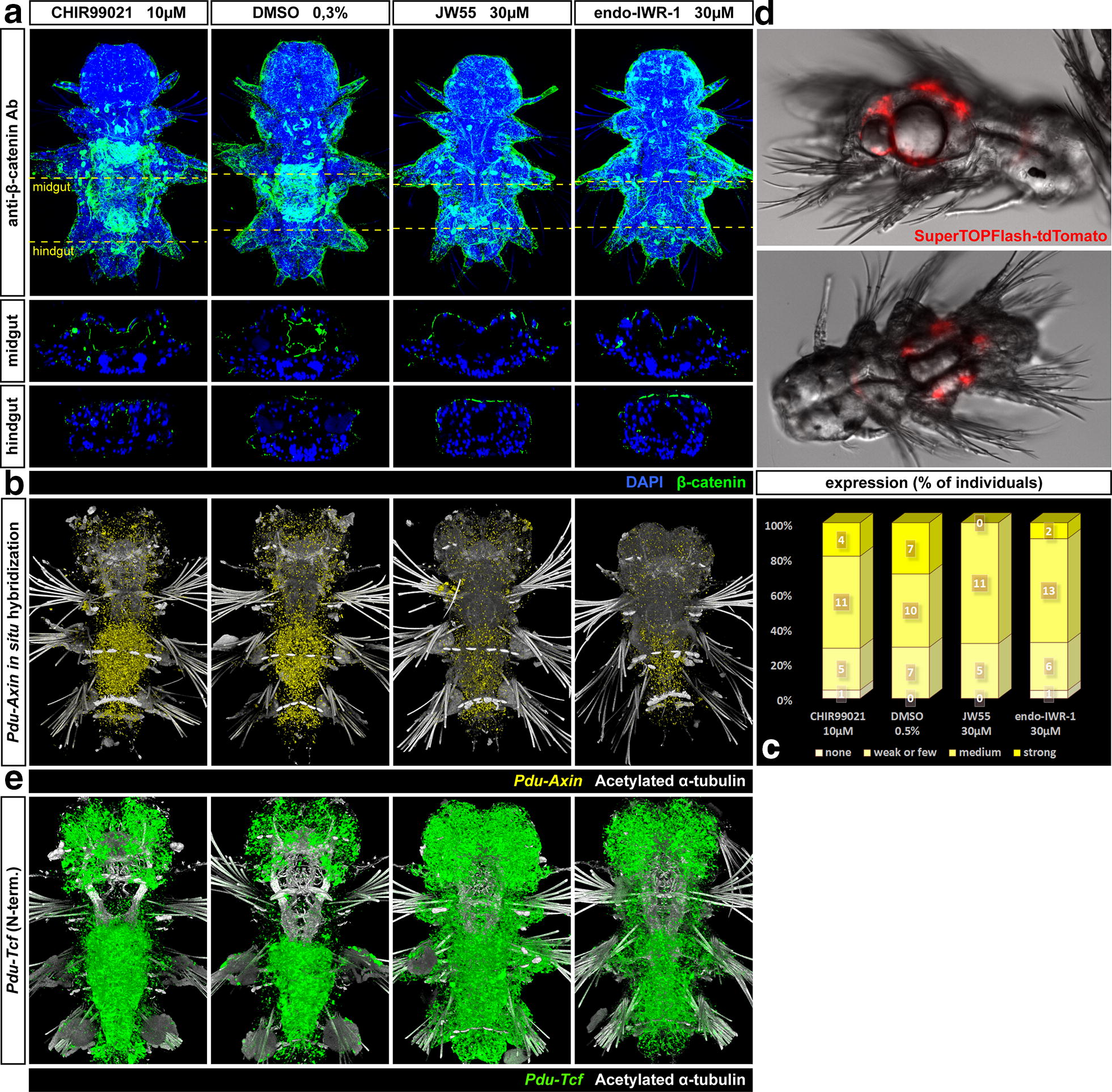
Wnt/β-catenin pathway is active in the larval gut and is affected by chemical manipulation. **a** Maximal projections and orthogonal virtual sections on the level of midgut and hindgut of a fluorescent confocal *z*-stacks of β-catenin protein immunostaining (green) on 7 dpf larvae done to confirm the chemical treatment’s efficacy. The larvae shown here come from the same batch as those used for in situ hybridization in Fig. 9. High levels of β-catenin were observed especially in the midgut. While the activation by CHIR99021 did not cause any dramatic increase in β-catenin levels, the inhibition of Wnt/β-catenin signalling by either of the inhibitors, JW55 or IWR-1-endo led to the complete absence of such high levels of β-catenin in the gut. **b** Fluorescent in situ hybridization of a putative Wnt target gene, *Pdu*-*Axin* (yellow) and quantification of the phenotype classes (**c**) shows no effect for the activator (CHIR99021) and mild effects of both Wnt/β-catenin inhibitors on axin expression. These larvae come from the same batch as those used for in situ hybridization in Fig. 10. **d** The fluorescent signal from the microinjected Wnt reporter construct SuperTOPFlash-tdTomato with 8 Tcf/LEF binding sites can be observed in the endoderm of 7 dpf transient transgenic larvae. This indicates that the Wnt/β-catenin signalling is active in the gut. **e** The effect of chemical manipulation of Wnt/β-catenin signalling on the expression of *Pdu*-*Tcf*

The presence of several components of the Wnt/β-catenin signalling pathway suggests that Wnt/β-catenin signalling is active in the larval gut. Besides, the expression of *Pdu*-*Tcf* transcription factor, a relatively high amount of β-catenin, the intracellular transducer of Wnt signal, was detected in the midgut, whereas it was much lower in the hindgut, although we were unable to distinguish nuclear and cytoplasmic staining (Fig. 7a). We also observed higher expression of the putative target gene *Pdu*-*Axin* in the midgut than hindgut and in the ring of ectodermal cells between the last segment and the pygidium (Fig. 7b).

To further support this evidence, we microinjected a SuperTOPFlash-tdTomato plasmid, which carries a gene for fluorescent protein under the promoter with eight repetitions of Tcf/LEF binding motif. The construct thus acts as a reporter of Wnt/β-catenin pathway activity [61]. We observed a red fluorescent signal in the gut of some transient mosaic transgenic larvae of *Platynereis* at 7 dpf (Fig. 7d), mainly in macromeres, where β-catenin is known to be stabilized from early development [39]. Thus, Wnt/β-catenin signalling is indeed active in the developing gut of *P. dumerilii* larvae.

### Pharmacological manipulation of Wnt/β-catenin pathway

To gain insights into the function of Wnt/β-catenin signalling in the *Platynereis* gut, we decided to chemically activate or inhibit the Wnt/β-catenin pathway in developing larvae from 5 dpf, when the *Pdu*-*Tcf* was still low in the midgut, to 7 dpf, when we observed the highest expression of *Pdu*-*Tcf*. To achieve this, we added chemical inhibitors or an activator of Wnt/β-catenin signalling to the sea water containing larvae. After chemical treatment, we fixed the larvae and observed differences in expression of gut-specific markers by in situ hybridization. Since after each chemical treatment, we often observed an array of phenotypes with different intensity and sometimes even a slightly variating pattern of staining, we attempted to quantify the results to measure the differences between the treatments by assigning every individual to categories according to their degree of marker expression. To ensure that the observed differences were due to the altered activity of Wnt/β-catenin signalling, we verified the efficacy of treatment by whole mount immunodetection of β-catenin (Fig. 7a) and by in situ hybridization of *Pdu*-*Axin* mRNA—Fig. 7b, c).

It has been previously shown that CHIR99021 activates Wnt/β-catenin signalling via the inhibition of GSK-3β, a part of the destruction complex, thus stabilizing β-catenin [66, 67]. JW55 has been shown previously to inhibit Wnt/β-catenin signalling in a cell reporter, gene expression and *Xenopus* double axis systems [68]. IWR-1-endo was previously demonstrated as a potent inhibitor of Wnt/β-catenin pathway [69] and has been used to inhibit Wnt/β-catenin signalling in *Platynereis* [46]. Both JW55 and IWR-1 function through the inhibition of tankyrase [68, 70]. We decided to use both inhibitors at a 30 µM final concentration, although 40 µM was used previously by others [46]. In our hands at 5–7 dpf stage, we saw an effect even with the 30 µM concentration; on the other hand, the higher concentration may have been lethal for the larvae. Our results suggest that IWR-1-endo was more potent and reliable Wnt inhibitor than JW55 and that it lowered β-catenin levels in the gut (Fig. 7a). On the other hand, JW55 seemed not to work as well on some batches; however when it did, both inhibitors yielded consistent results. Interestingly, none of the inhibitors caused the complete loss of expression of a putative Wnt target gene *Pdu*-*Axin*. This might be attributed to the possibility that *Pdu*-*Axin* is only partially regulated by Wnt/β-catenin signalling (for details, see “Discussion” section). Although tankyrase inhibitors act through the stabilization of Axin, this happens on the protein level and should not affect mRNA. We were interested to see whether *Pdu*-*Tcf* itself was a target of Wnt/β-catenin. There appear to be fewer *Pdu*-*Tcf* transcripts upon Wnt/β-catenin inhibition and more upon activation in the gut, which suggests a positive feedback loop, whereas the opposite is the case for the rest of the body. However, these conclusions are based on a single observation and the effect was not quantified.

### Wnt/β-catenin signalling regulates proliferation

Wnt/β-catenin signalling in general induces cell proliferation [71], and in vertebrate gut, Wnt activity at the base of crypts maintains cells in a proliferative state. Therefore, we were curious, whether a similar effect on cell proliferation in *Platynereis dumerilii* could be observed as well. We performed chemical activation and inhibition treatments from 5 to 7 dpf as described above, but this time we added the nucleotide analogue 5-ethynyl-2′-deoxyuridine (EdU), which is incorporated into replicating DNA in vivo, to the water containing 6 dpf larvae. EdU contains an ethynyl group that after activation is later labelled with a fluorescent dye to visualize proliferating cells [72].

**Fig. 8:**
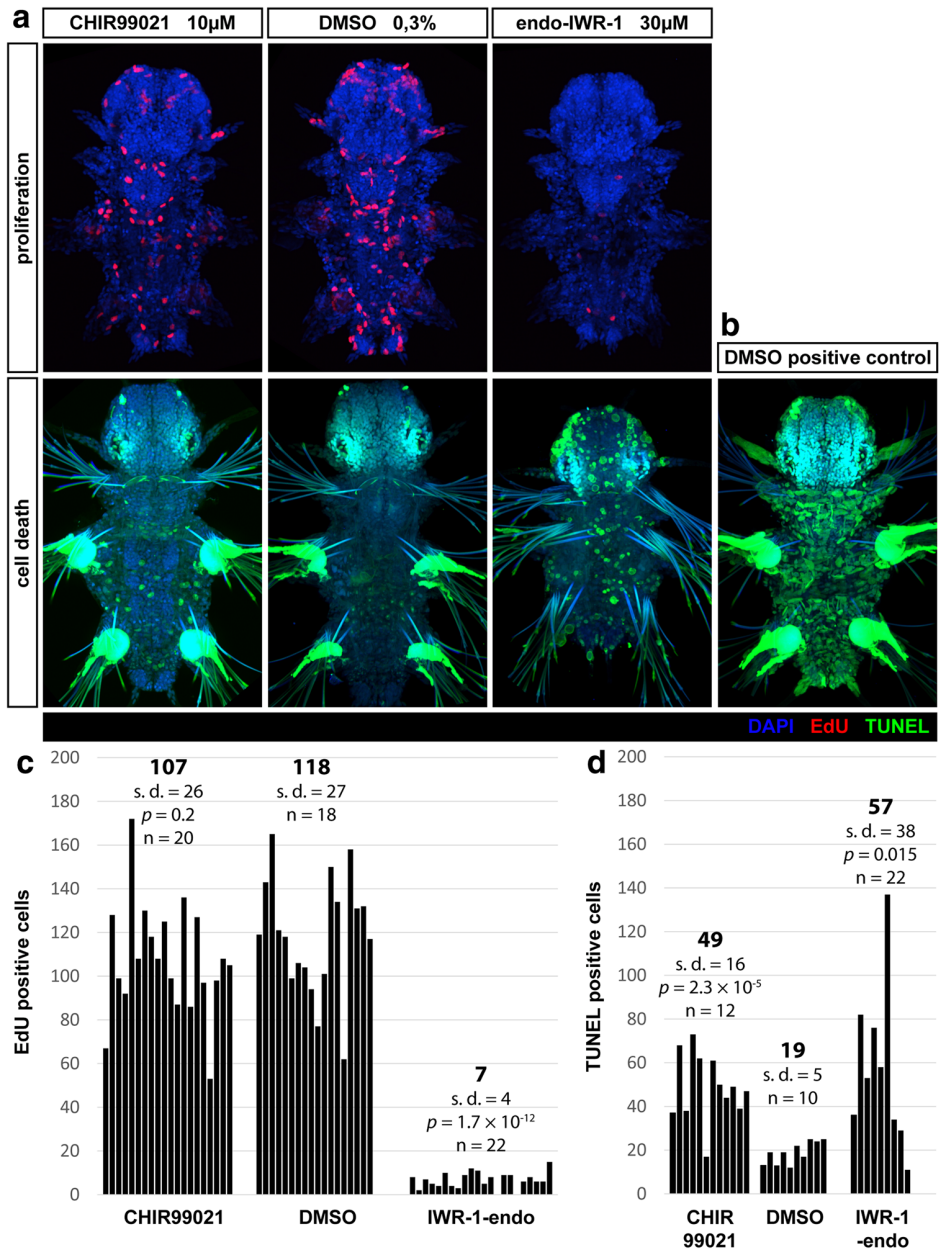
Wnt/β-catenin signalling is necessary for cell proliferation and survival. **a** Proliferating cells labelled by incorporation of 5-ethynyl-2′-deoxyuridine (EdU, red) from 6 to 7 dpf and counterstained with DAPI (blue) to mark cell nuclei. Images are maximal projections of the whole fluorescent confocal *z*-stacks. Representative individuals are shown. **b** TUNEL staining of cell death (green), counterstained with Hoechst dye (blue) to mark nuclei. **c** Number of EdU positive proliferating cells in the whole larvae as counted manually on maximal projections of fluorescent *z*-stacks as those shown in (**a**). Averages are indicated in bold, *n*—number of individuals analysed per each experimental group, *p*—*p* value of a standard two tailed unpaired Student’s t test with unequal variance, *s.d.*—standard deviation. There are significantly less proliferating cells when IWR-1-endo is present. With the activator of Wnt/β-catenin signalling CHIR99021, there is no significant difference in the number of proliferating cells throughout the body. **d** Quantification of cell death performed in the same way as for proliferation, except only cells from the trunk up to midgut/pharynx boundary (or up to first pair of parapodia) were counted. There are significantly more dead or dying cells in groups treated with CHIR99021 or IWR-1-endo compared to control group which was treated with DMSO

In *Platynereis* at 7 dpf, most of the proliferating cells were located in the frontal part of the head and in the proliferative zone between the last segment and pygidium, surrounding the hindgut (Fig. 8a). Some cells could be seen in pygidium itself at the base of anal cirri and around anus (potentially identical to *Pdu*-*Otx*-expressing cells, see further), at the base of second and third pair of parapodia, the jaws and on the foregut/midgut and midgut/hindgut borders. Only few proliferating cells were seen on the ventral side of midgut.

Overall, there were significantly less proliferating cells throughout the body of IWR-1-endo treated larvae, whereas the number of proliferating cells in the presence of Wnt activator CHIR99021 was not changed (Fig. 8c). Thus, it seems that active Wnt/β-catenin signalling is required for cell proliferation.

We also checked for cell death by TUNEL staining of fragmented DNA. There were significantly more dead or dying cells in the larvae treated with CHIR99021 or IWR-1-endo. However, this test does not discriminate between apoptosis and other forms of cell death. Since most dead cells are present on the surface of the body, which is in direct contact with the environment, and the dead cells are seen in the presence of either activator or inhibitor, the cell death cannot account for the observed changes in gene expression inside the gut which are different for each chemical. Moreover, expression of some gut marker genes shows that a morphologically normal gut was present in all experimental groups.

### Downregulation of Wnt/β-catenin pathway converts midgut to hindgut

*Platynereis* gut consists of three major parts, a foregut, a midgut and a hindgut. To visualize the effect of chemical manipulation of the Wnt/β-catenin pathway on gut differentiation, we used the expression of several digestive enzymes that have been previously described to be specific to different *Platynereis* gut compartments [41]: extracellular peptidases *Pdu*-*Subtilisin*-*1* and *Pdu*-*Subtilisin*-*2*, a polysaccharide digesting enzyme *Pdu*-*α*-*Amylase* and a precursor of an intracellular digestive protease *Pdu*-*Legumain*.

**Fig. 9:**
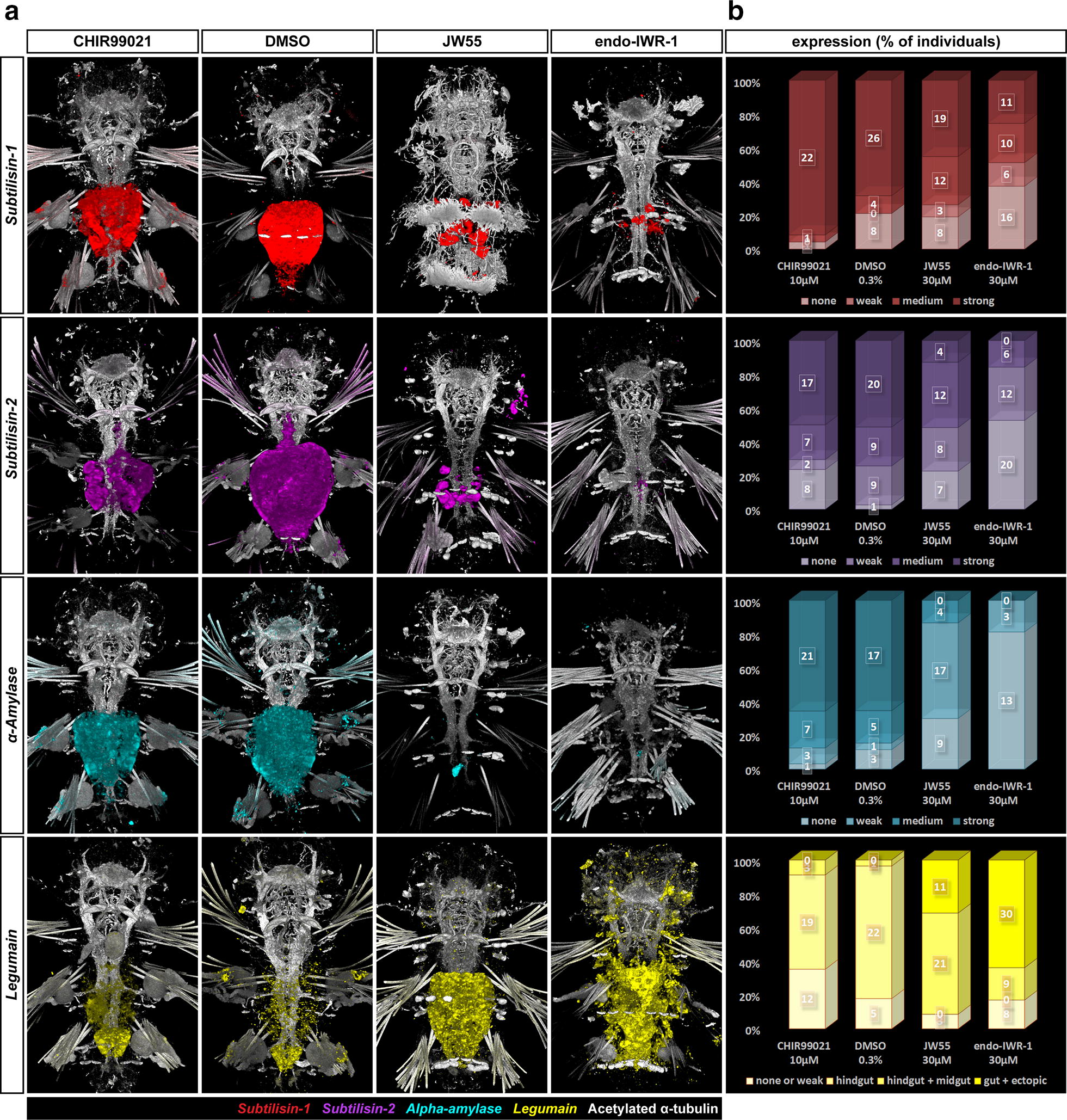
Inhibition of Wnt/β-catenin signalling converts the midgut to hindgut. **a** Fluorescent in situ hybridization of gut marker genes. **b** Quantification of the effect of activation/inhibition of Wnt/β-catenin pathway on the expression of these genes by assignment of all individuals to phenotypic classes. *Pdu*-*Subtilisin*-*1* (first row, red)*, Pdu*-*Subtilisin*-*2* (second row, magenta)*, Pdu*-*α*-*Amylase* (third row, cyan) = midgut marker genes, the darker the colour in the graph, the higher the expression. All three midgut marker genes show remarkably lower or no expression when Wnt/β-catenin signalling is inhibited (JW55 or IWR-1-endo). No pronounced effect was observed for activation of the pathway (CHIR99021). *Pdu*-*Legumain* (fourth row, yellow) = hindgut marker gene is normally highly expressed in the hindgut but only in very low levels in the midgut, which is also the case in the presence of activator, but it expands to midgut upon Wnt/β-catenin inhibition. Moreover, stronger inhibition (IWR-1-endo) causes it to be expressed outside the digestive tract in nephridia and other cells

*Pdu*-*Subtilisin*-*1* showed only faint expression in the hindgut and was primarily expressed in the midgut (Fig. 9, top row), similar to *Pdu*-*Subtilisin*-*2* (Fig. 9, second row) and *Pdu*-*α*-*Amylase* (Fig. 9, third row). All three of these genes thus exhibited predominantly midgut expression (in the latter two consistently with the pattern described by Williams et al. [41]). We observed much stronger expression of *Pdu*-*Legumain* protease precursor in the hindgut than in the midgut, making *Pdu*-*Legumain* a useful hindgut marker gene. The results of quantification are summarized in Fig. 9b. Genes expressed in the midgut, but not in the hindgut at 7 dpf (*Pdu*-*Subtilisin*-*1*, *Pdu*-*Subtilisin*-*2*, *Pdu*-*α*-*Amylase*) were expressed at lower levels or diminished in the midgut upon Wnt/β-catenin inhibition (Fig. 9, top, second and third rows), while the hindgut-specific gene *Pdu*-*Legumain* (Fig. 9, bottom row) expanded from the hindgut to midgut and even outside gut to nephridia (compare with [65]) under the same conditions. The midgut thus obtains hindgut-like characteristics when the Wnt/β-catenin signalling is inhibited. Activation of the Wnt/β-catenin pathway by CHIR99021 had no major effects on gene expression, which might reflect that Wnt/β-catenin signalling was already active in the midgut, where it is required, but not sufficient, to trigger midgut fate.

**Fig. 10:**
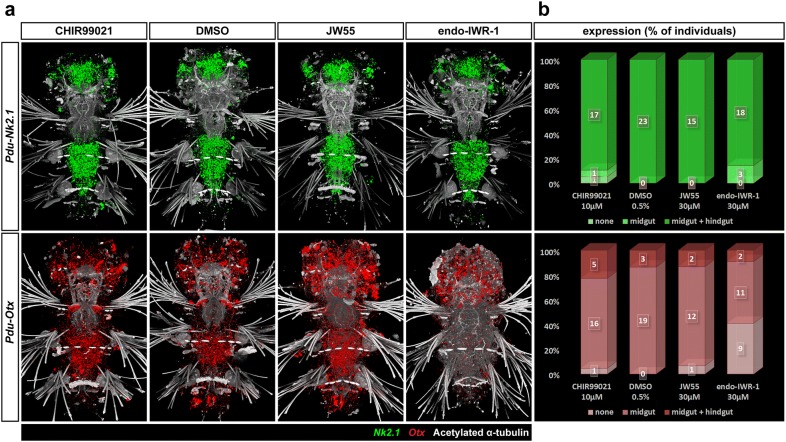
Expression of endodermal marker genes. **a** Fluorescent in situ hybridization of two endodermal marker genes, *Pdu*-*Nk2.1* and *Pdu*-*Otx* and **b** quantification of the effect of activation/inhibition of Wnt/β-catenin pathway on their expression by assignment of all individuals to phenotypic classes. The expression of these genes is typical for neuroectoderm in early larvae but similar to *Pdu*-*Tcf*, they obtain new expression domains in the gut later in development while still retaining their neurospecific expression. *Pdu*-*Nk2.1* (top row, green) is expressed in both midgut and hindgut under normal circumstances, and the same pattern is observed in the presence of any of the tested inhibitors or the activator. This is consistent with the theory of midgut to hindgut conversion upon Wnt/β-catenin signalling inhibition since the gene is normally expressed in both tissues. *Pdu*-*Otx* is typical for mandibular cells, the entire midgut, whereas it is absent from most of the hindgut except for the very posterior cells which form the sides of the anus. Its midgut expression disappears in the presence of the stronger inhibitor (IWR-1-endo), while a normal pattern is observed with the milder JW55 inhibition (which might just point to unsuccessful inhibition on this batch). Neural and mandibular expression is preserved under all conditions, although it is somewhat lower after Wnt/β-catenin inhibition given the overall smaller size of mandibles. Interestingly, the cells surrounding the anus lose *Pdu*-*Otx* expression also in the presence of either CHIR99021 activator of Wnt/β-catenin signalling

We also looked at the expression of two endomesodermal genes, *Pdu*-*Nk2.1* and *Pdu*-*Otx*. They are typical for the neuroectoderm early in its development [73–76], but *Otx* has also been documented in stomodaeum [55, 75]. However, both genes are known to be important regulators of endomesodermal and gut development of Bilateria, e.g. starfish [77], and even Cnidaria [78]. In addition, both were also found in the developing gut of other annelids—*Capitella teleta* [79] or (in the case of *Lox10* gene, a plausible *nk2* homologue) the leech *Hellobdela triseralis* [80]. In 7 dpf *Platynereis*, *Pdu*-*Nk2.1* (Fig. 10, top row) was expressed in a large domain in the middle of head between two stems of axons protruding anteriorly from the brain and also in two smaller domains on sides of the head, laterally from these axonal bundles in the eye region. In the gut, similarly high levels were observed in both midgut and hindgut, which is consistent with what has been published for *Capitella* [79]. *Pdu*-*Otx* transcripts were found throughout the head, more abundant in the domains that were medially adjacent to the forementioned axonal bundles, the small ectodermal patches anterior from these axons, and on the posterior–lateral sides of the head. In the digestive tract, *Pdu*-*Otx* (Fig. 10, bottom row) was expressed in the jaws, in the midgut, but not hindgut, and strongly in two cells surrounding the anus. It diminishes from the midgut upon the inhibition of Wnt/β-catenin pathway by IWR-1-endo, similar to midgut-specific enzymes. The loss of *Pdu*-*Otx* expression from the two anal cells in the presence of either activator or inhibitor suggests that these cells require a precisely regulated activity of the Wnt/β-catenin signalling to activate *Pdu*-*Otx*. *Pdu*-*Nk2.1*, which is normally expressed in both the midgut and hindgut, does not change its expression pattern upon either activation or inhibition.

**Fig. 11:**
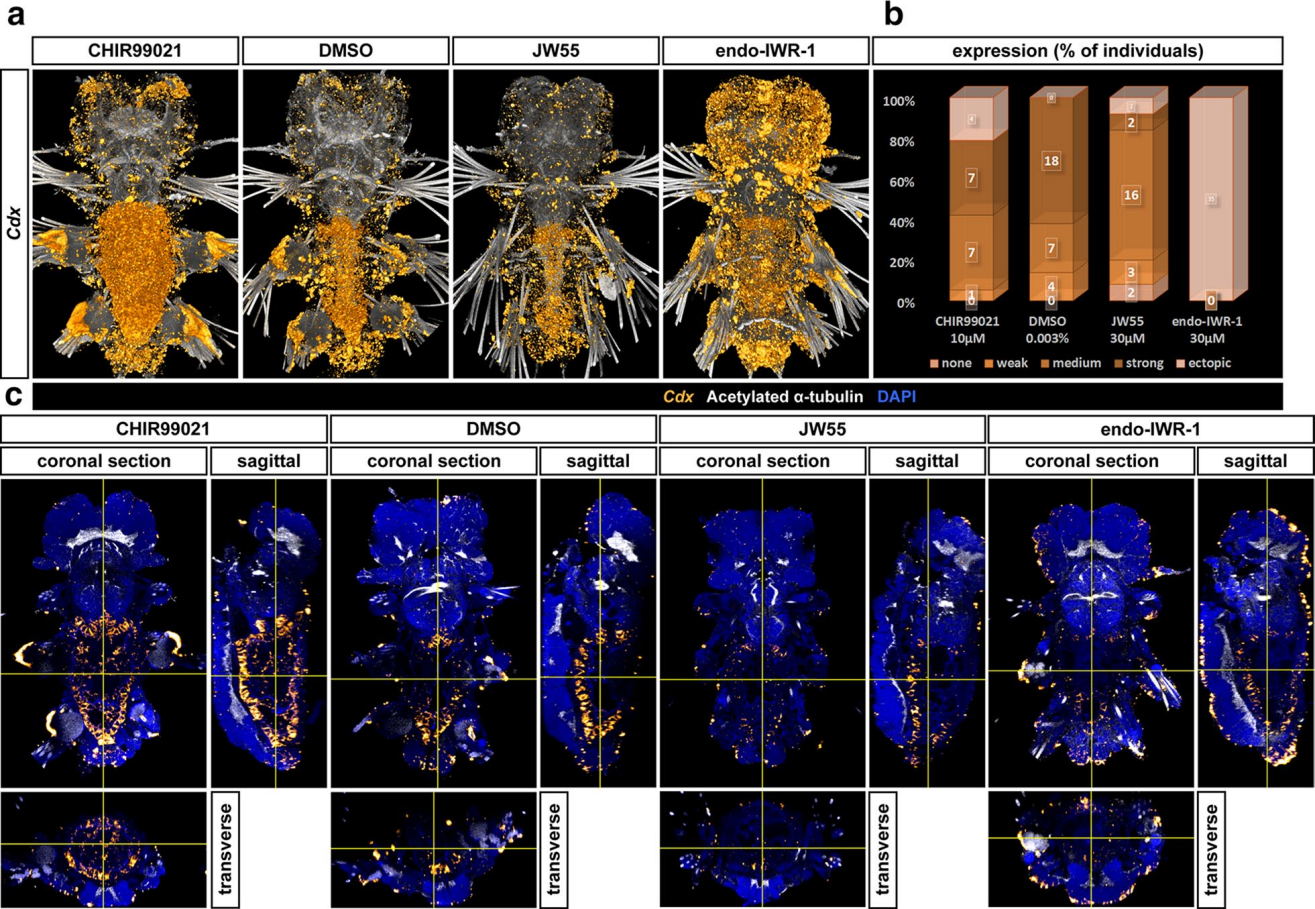
Expression of *Pdu*-*Cdx* ParaHox gene. **a** 3D reconstructions of confocal fluorescent in situ hybridization’s *z*-stacks detecting a ParaHox gene *Pdu*-*Cdx* and **b** A graph showing the percentage of individuals assigned to classes according to the level of expression (the darker the higher) upon Wnt chemical treatment. Under control conditions, *Pdu*-*Cdx* is expressed in the whole hindgut, (in lower level) on the ventral floor of midgut and on the foregut/midgut border. With the Wnt activator (CHIR99021), it was expressed in similar levels but in entire gut, whereas with the inhibitor JW55, the expression is somewhat lower but the pattern remains the same. Widespread ectopic expression with the stronger inhibitor (IWR-1-endo) prevented the proper quantification of phenotypes when using bright-field microscopy. **c** Orthogonal views of the same *z*-stacks reveal the expression in the hindgut and at the midgut/foregut boundary connected by a narrow strip of *Pdu*-*Cdx* expression on the ventral floor of the gut. This expression expands to most of the gut upon Wnt activation, and the overall expression is lower in the presence of Wnt inhibitors

**Fig. 12:**
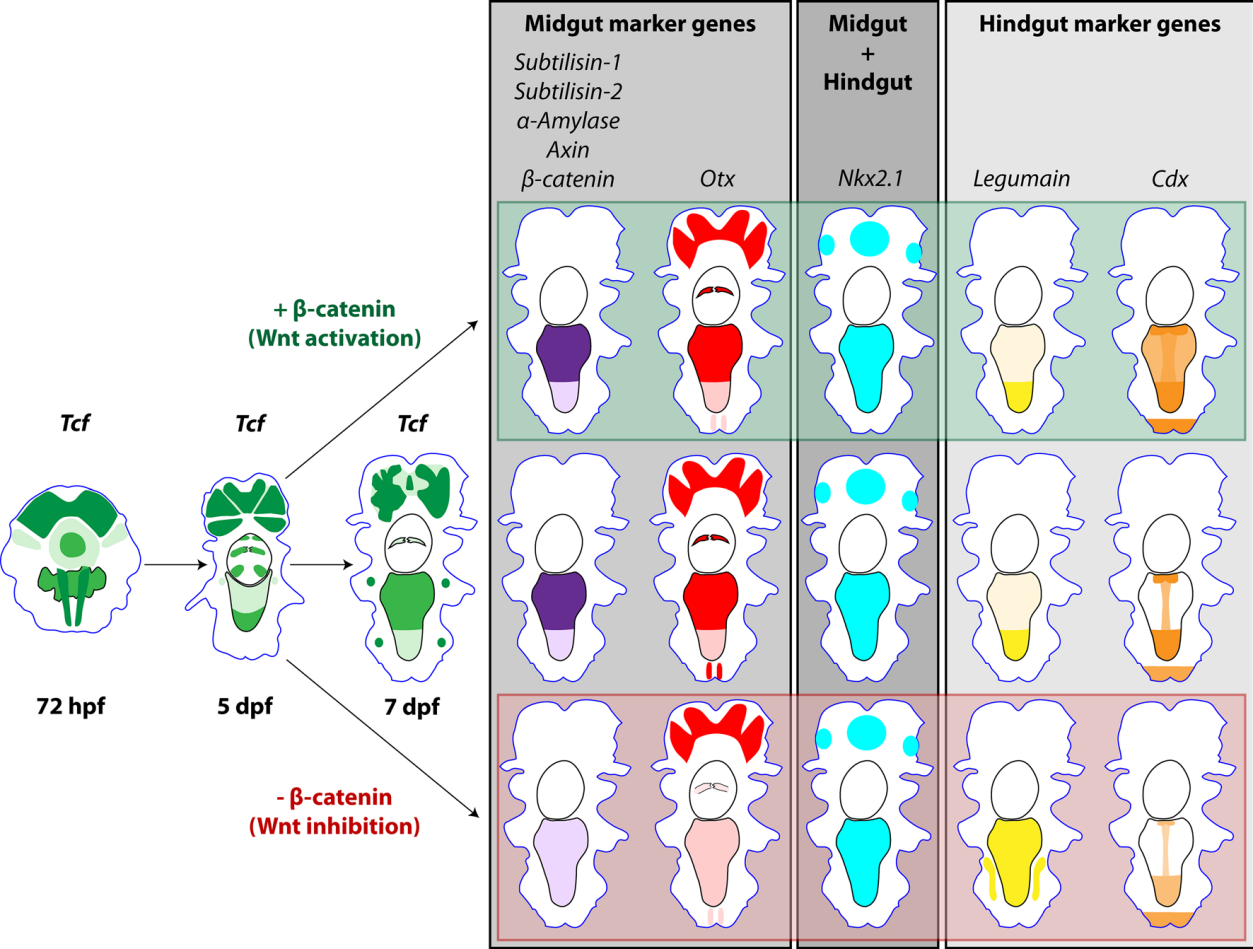
Schematic representation of *Pdu*-*Tcf* expression and the role of Wnt/β-catenin signalling in gut development. This diagram summarizes the expression of *Pdu*-*Tcf* from the 72 hpf stage with putative gut primordium, through 5 dpf stage with uncellularized gut to 7 dpf larva with a functional, compartmentalized digestive tract. Midgut marker genes are generally downregulated by inhibition of Wnt/β-catenin signalling, whereas hindgut marker gene *Pdu*-*Legumain* expands to midgut. The expression profile of these genes in midgut is thus more reminiscent of the hindgut upon Wnt inhibition. On the other hand, another hindgut gene *Cdx* is slightly downregulated upon inhibition. We did not observe any pronounce effect of Wnt/β-catenin pathway activation, except for *Cdx* which is upregulated in the midgut. The expression of the endodermal marker, *Nk2.1*, which is present in both compartments under normal circumstances, is not changed by either manipulations of the Wnt/β-catenin pathway. According to our scenario, Wnt/β-catenin signalling is necessary for endoderm to differentiate into midgut secretory epithelium and its inhibition causes midgut cells to be functionally converted to the hindgut

The role of the *Cdx* gene in hindgut formation has been well documented in vertebrates (reviewed in [5]), sea urchins [81], ascidians [82], *Drosophila* [12] and other organisms. It is directly activated by Wnt/β-catenin signalling via Tcf4 [8], or it can conversely trigger Wnt expression as seen in the sea urchin [81]. *Pdu*-*Cdx* is also expressed in the hindgut of *Platynereis* [43, 56] and *Capitella* [83]. We used in situ hybridization of a *Pdu*-*Cdx* probe to detect its expression after chemical treatment of Wnt/β-catenin signalling. In controls, *Pdu*-*Cdx* was expressed in the hindgut and the foregut/midgut boundary, with these two domains connected by a weaker expression on the ventral side of the midgut (Fig. 11a), close to sources of Wnt and consistent with the notion that *Cdx* genes require a high Wnt signal for activation. Interestingly, *Pdu*-*Cdx* expands from the ventral side through the entire midgut upon Wnt activation in some individuals but was expressed in a normal pattern, though at somewhat lower levels, when the pathway is inhibited (Fig. 11). The changes in expression of all studied genes are summarized in Fig. 12.

## Discussion

### *Pdu-Tcf* isoforms

It was previously pointed out that a great difference exists between the diversity and specialized functions of vertebrate Tcf proteins with their many isoforms in contrast to the poor *Tcf* gene repertoire in invertebrates (both deuterostomes and protostomes) [16, 21]. Their genomes, with some exceptions (Planaria, Platyhelminthes), usually comprise only one *Tcf* gene, and their repertoire of isoforms has been underappreciated until recently, although their presence in other organisms as *Drosophila* has also been mentioned. Thus, it is not clear whether complex regulation by Tcf proteins is limited only to organisms that possess multiple *Tcf/LEF* genes or whether it is also present in Protostomia with only single *Tcf* gene, and if yes, how it is achieved. Here, we have shown that a large diversity of Tcf isoforms is present in a spiralian which can potentially compensate for the lack of gene diversity in invertebrates. According to our BLAST search, *Tcf* isoforms have been already found in the sequencing of many invertebrate genomes. From vertebrates, the top hits usually belonged to various isoforms of the *Tcf7l2* (*Tcf4*) gene. Since the duplications of *Tcf* genes occurred only after the divergence of *Platynereis* and vertebrate lineages, this does not reflect closer homology than with other vertebrate *Tcf* genes, but it could rather point out to closest functional similarity. It is interesting that *Tcf4* is able to both activate [30] and repress [29] transcription depending on context [28] and it is necessary for renewal of gut epithelium [30], while *Tcf1* and *Tcf3* are considered to be purely an activator or an inhibitor of gene expression, respectively [27]. We hypothesize that Pdu-Tcf is able to undertake both the role of activator and repressor function. In addition, we propose that individual Tcf isoforms may have slightly different DNA binding characteristics and hence select overlapping, but non-identical sets of target genes and activates or represses them with a different strength relative to each other than another isoform.

### Wnt/β-catenin signalling in gut development

The conservation of Wnt/β-catenin signalling’s role in digestive tract development still remains questionable due to a lack of detailed data from mostly invertebrate phyla, namely the group Spiralia, which do not contain any of the “classical” model organisms. Cell lineage tracing and fate mapping have been done several times in *Platynereis* in an increasingly greater depth, most recently up to the midtrochophore stage. Expression profiling based on single-cell RNA seq, back-mapping to the larval body based on a reference gene expression atlas and subsequent clustering according to the typical expression fingerprints of major organ systems [84] revealed cell populations in early larva which later grow and differentiate to the gut. It presumably originated from two small clusters of peptidergic cells that express *Hnf4* and symmetrically positioned along the ventral midline on the level between second and third segment. Since *Pdu*-*Tcf* is expressed broadly in putative endodermal and mesodermal cells in the body of the 48 hpf larva, it is probable that it is expressed in *Hnf4-*positive cells as well. Moreover, *Pdu*-*Tcf* is present in internal clusters of cells at 72 hpf reminiscent of *Hnf4*+ putative gut precursor cells and might thus represent the developing gut. Hence, it is possible that *Pdu*-*Tcf* is expressed in the gut from the earliest stages of its development.

Later in development, *Pdu*-*Tcf* transcripts can be detected in the hindgut and two paired domains (one of them comprising the jaws) in the foregut, but only slightly in the midgut of a 5 dpf larva. This may possibly be due to the fact that the midgut at this stage consists primarily of large not yet cellularized macromeres. Interestingly, this pattern corresponds to the expression of *Tcf4* and *Lef1* in the developing chicken gut, where they are necessary for formation of gizzard microvilli and differentiation of hindgut cells, respectively. However, in the cellularized gut of the 7 dpf larva, *Tcf* is already strongly transcribed in the midgut but less in hindgut evoking more the expression of *Tcf4* in both developing and adult mammalian gut [26, 85]. Not surprisingly, given that *Pdu*-*Tcf* is expressed in the developing jaws, it is of note that the chitinous mandibles of treated larvae are smaller and less developed. Together with our finding that cell proliferation in the gut (and throughout the whole body) is severely reduced or abolished in larvae treated with a strong Wnt inhibitor, these results suggest that Wnt/β-catenin signalling is necessary for midgut cellularization which requires cell division, reminiscent of the *Tcf4* role in the maintenance of mammalian adult gut epithelium [30].

Expression of endodermal marker gene *Pdu*-*Nk2.1* shows that the midgut cells of larvae, whose Wnt/β-catenin signalling was inhibited, retained endodermal characteristics. Yet, the midgut marker genes *Pdu*-*Subtilisin*-*1*, *Pdu*-*Subtilisin*-*2*, *Pdu*-*α*-*Amylase* and *Pdu*-*Otx*, which are under normal circumstances expressed at high levels in the midgut but absent from the hindgut at 7 dpf, are lost upon pharmacological inhibition of Wnt/β-catenin pathway. At the same time, the hindgut marker gene *legumain* protease precursor expands from hindgut to midgut. Taken together, these two pieces of evidence suggest that midgut cells lose their ability to express digestive enzymes and obtain (or retain) hindgut-like characteristics, when Wnt/β-catenin is inhibited. All changes in gene expression are summarized in the scheme illustrated in Fig. 12.

There is small, but a notable difference between the expression patterns of digestive enzymes reported in our paper and in previous work conducted by Jékely group [41] which reported that *Pdu*-*Subtilisin*-*1* and *Pdu*-*Legumain* are expressed in both the midgut and the hindgut, whereas we found that *Pdu*-*Subtilisin*-*1* was predominantly a midgut gene and *Pdu*-*Legumain* was expressed strongly in the hindgut but only very weakly in the midgut (although some inter-individual variance among larvae is already present at this life stage and some individuals with expression in both gut compartments can be found). The likely explanation for this discrepancy comes from the fact that Williams and colleagues described the expression on 6 dpf stage, when cellularization of the gut is still occurring and the differentiation of the gut has not been completed. Initially, the midgut and hindgut cells are more alike, and only at 7 dpf stage, the expression patterns become more resolved with most of the digestive enzymes expressed in the broad midgut, while the narrow hindgut is dedicated more to defecation than digestion.

*Pdu*-*Cdx* can also be considered a hindgut gene, but unlike *Pdu*-*Legumain,* we saw quite the opposite effect on its expression, which was downregulated upon Wnt inhibition and upregulated in the midgut in the presence of a Wnt activator. *Pdu*-*Cdx* is a direct target that is activated by Wnt/β-catenin signalling [8]. On the other hand, the digestive enzymes are probably indirect targets which can be upregulated by the decrease in their transcriptional repressors, themselves under the control of Wnt/β-catenin signalling, and can thus react to Wnt inhibition opposite than *Cdx*. The transcription of *Cdx* genes is fully triggered only by Tcf proteins that have a functional, intact C-clamp [36]. Although we do not know which *Pdu*-*Tcf* isoform is expressed in the gut, the involvement of a C-clamp (−) isoform might explain why *Pdu*-*Cdx* transcription is not activated in the midgut under normal circumstances, with the exception of the ventral floor and both foregut and hindgut borders that are closest to Wnt sources.

The observation that chemical manipulation of the Wnt/β-catenin pathway from 5 to 7 dpf does affect the expression of the Wnt target gene *Pdu*-*Axin*, but not dramatically, although reported to be the case at 55 dpf [46], which may be explained by the fact that vertebrates have two *Axin* genes and only *Axin2,* but not *Axin,* has been shown to be regulated by Wnt/β-catenin signalling [17] even though they are functionally equivalent in vivo [86]. On the other hand, *Platynereis* has most probably only one *axin* gene, whose responsiveness to Wnt/β-catenin signalling might be time and tissue dependent. Immunostaining against β-catenin clearly shows that the stronger inhibitor IWR-1-endo indeed reliably downregulates Wnt/β-catenin signalling and β-catenin staining and the effects on gene expression shared with IWR-1-endo suggest that the other inhibitor, JW55, works as well but produce milder phenotypes at the same concentration. The reliability of the activator CHIR99021 remains questionable since it always produced mild or no effects at this stage (except the missing *Pdu*-*Otx*-positive cells of the anus) although it works very well on younger larvae (our observation, data not shown). The absence of strong phenotypic and gene expression differences in the presence of the activator can also suggest that the Wnt/β-catenin signalling has a permissive instead of an instructive role in gut development. In such a scenario, the pathway’s activity is required, but not sufficient for the gut endoderm to acquire midgut fate and gene expression fingerprint. Therefore, other transcriptions factors and signalling pathways would have to be involved to pattern the developing gut as has been shown for Hox genes [87], Hedgehog [88, 89], BMP [90] and Notch signalling [91] in other organisms.

Based on preliminary results, it seems that *Pdu*-*Tcf* itself might be a target of Wnt/β-catenin pathway. Upon Wnt activation, it is strongly transcribed in both the midgut and the hindgut, whereas the expression is lower after inhibition. This points to a positive feedback loop to reinforce *Pdu*-*Tcf* expression in the gut, where high amounts might be necessary. Positive autoregulation has been described before for LEF1 in colon cancer [92], XTcf-4 (Tcf7l2) in *Xenopus* midbrain [93] and zebrafish Tcf3 (Tcf7l1) [95]. It is achieved through the direct binding of Tcf to its promoter [94, 96], but, in vertebrates, this can be mediated by a different Tcf [97]. Interestingly, there was higher and broader *Pdu*-*Tcf* expression in the presence of Wnt/β-catenin inhibitors in the rest of the body, e.g. brain ganglia. This could be explained by the presence of a negative feedback loop to maintain steady and localized *Pdu*-*Tcf* expression. Such dual regulation in two different tissues, if confirmed, could be achieved by the action of tissue-specific sets of transcriptional cofactors. Further investigation will be needed to verify these results and ascertain the real situation about *Pdu*-*Tcf* autoregulation.

The differences between the midgut and the hindgut and the observed midgut to hindgut transition caused by inhibition of Wnt signalling (Fig. 12) probably cannot be accounted for *Pdu*-*Tcf* alone. Our in situ hybridization stainings show that it is expressed in the hindgut and some parts of foregut at 5 dpf, whereas it is present in both midgut and less in hindgut later at 7 dpf. The same is true for *Pdu*-*Axin* at 7 dpf. If we take a look at immunofluorescence staining of β-catenin as a proxy of Wnt/β-catenin pathway activity, we see it is present in high amounts in the midgut but not in the hindgut. This difference might be the results of the combinatorial action of factors involved in Wnt/β-catenin signalling cascade or in the modulation of Wnt signal, e.g. sources of Wnt proteins, expression of Frizzled receptors or soluble Wnt inhibitory proteins. When the Wnt/β-catenin pathway is inhibited, β-catenin diminishes from the midgut, whereas no change occurs in the hindgut, since β-catenin levels there are naturally low. As a result, the gene expression regulated by Wnt signalling via β-catenin in both parts of the gut exhibits hindgut-like characteristics.

## Conclusions

In this paper, we described a single *Tcf* homologue in the marine polychaete annelid *Platynereis dumerilii*. It produces an array of mRNA variants via alternative splicing with a potentially different DNA binding capacity and function. *Pdu*-*Tcf* and several other components of Wnt/β-catenin signalling pathway (*Axin*, β-catenin) are present in the developing gut of *Platynereis* and inhibition of Wnt/β-catenin signalling causes the midgut to obtain a hindgut-like expression of digestive enzymes and leads to a loss in cell proliferation. Taken together, *Platynereis* appears to be a useful model to investigate the roles of Wnt/β-catenin signalling in organ development in a relatively simple system and to find features of gut differentiation and maintenance that have been conserved in Bilateria.
